# The impact of population's educational composition on Healthy Life Years: An empirical illustration of 16 European countries

**DOI:** 10.1016/j.ssmph.2021.100857

**Published:** 2021-06-26

**Authors:** Markus Sauerberg

**Affiliations:** Vienna Institute of Demography (OeAW), Wittgenstein Centre for Demography and Global Human Capital (IIASA, OeAW, University of Vienna), Vordere Zollamtsstrasse 3, 1030, Vienna, Austria

**Keywords:** Health, Mortality, Education, Inequality, Composition, GALI, Healthy Life Years, Life expectancy, Europe

## Abstract

Healthy Life Years (HLY) is a prominent summary indicator for evaluating and comparing the levels of population health status across Europe. Variations in HLY, however, do not necessarily reflect underlying differences in health and mortality levels among countries and the indicator is particularly sensitive when broken down by subpopulations. For instance, despite European countries showing large HLY inequalities by educational level, these countries are also heterogenous regarding their population composition by educational attainment, which most likely affects their HLY levels. We demonstrate how this compositional effect shapes HLY levels by providing estimates for HLY by educational attainment and gender for 16 European countries using the Sullivan method. We use prevalence data about limitations in daily activities from the European Union Statistics on Income and Living Conditions (EU-SILC) and mortality data from the Eurostat database. Finally, we adjust for compositional effects by means of standardization. The education-adjusted HLY estimates do not differ much from conventional HLY. Yet, we find that in some countries HLY levels are indeed affected by the population composition by educational attainment. For example, low-, medium-, and high educated individuals in Portugal show more HLY than their counterparts in Poland. Still, Poland's total HLY value slightly exceeds that of Portugal, indicating favorable health and mortality conditions in Poland. It is Poland's lower relative number of low educated individuals in its population that is responsible for producing this higher total HLY value. We conclude that differentials in HLY due to differences in the relative size of educational subpopulations are generally small in HLY across Europe but they can play an important role for countries that experienced large differences in their educational expansion.

## Highlights

• Providing Healthy Life Years estimates by educational attainment for 16 European countries.
• Revealing large educational inequalities in Healthy Life Years across Europe.
• Illustrating the impact of population's educational composition on Healthy Life Years in Europe.

## 1 Introduction

Health and mortality are well-documented as being strongly associated with socioeconomic factors, with individuals of higher socioeconomic status living longer and healthier lives than persons of lower socioeconomic status in both developed and developing countries (Mackenbach et al., 2008, Mackenbach et al., 2017; Preston & Taubman, 1994; Beltrán-Sánchez & Andrade, 2013). An individual's socioeconomic status can be measured through a multiplicity of factors, including income, wealth, education, and occupation. However, research consistently shows that, net of these other factors, educational attainment plays a prominent role in shaping health outcomes, since more educated individuals tend to avoid risky health behaviors (Brønnum-Hansen & Juel, 2004; Cai et al., 2010; Pampel et al., 2010; Singer et al., 2019) and are among the first to adopt and have access to medical technologies (Link and Phelan, 1995; Glied & Lleras-Muney, 2008). Studies also demonstrate a strong educational gradient in both longevity and mortality compression with higher educated individuals living longer lives and experiencing less lifespan variation (Van Raalte et al., 2011). This evidence suggests that the relationship between education and population health might not only refer to simple correlations, but rather reflect a causal mechanism in which higher education directly translates into better population health through individual behavior and increased social capacity for population health (Davies et al., 2018; Brunello et al., 2015; Lutz & Kebede, 2018; Fogel & Costa, 1997). In addition, among all the socioeconomic factors, educational attainment has been identified as the single most important source of observable population heterogeneity that should be routinely added in any demographic analysis (Lutz & KC, 2011). Consequently, research has consistently found substantial differences in terms of life expectancy at age *x* ($e_{x}$) as well as in the Healthy Life Years (HLY) between educational subpopulations (Majer et al., 2010; Mäki et al., 2013; Rubio-Valverde et al., 2021), with educational inequalities in HLY surpassing those of $e_{x}$ (see Cambois et al., 2020 for a detailed literature review).

Indicators like $e_{x}$ and HLY are well-known tools for assessing population health in Europe. While $e_{x}$ is a pure mortality indicator that reflects the expected number of *total life years*, HLY combines information on health and mortality to measure the expected number of *healthy life years* (Mathers, 2002). In light of aging populations, HLY has become an increasingly relevant indicator of population health and sustainability level (Lutz, O'Neill and Scherbov 2001; Christensen et al., 2009). It directs health policies and measures health gaps between countries (Murray et al., 2000). However, HLY indicators can be problematic for cross-country comparisons due to several reasons. The imperfect harmonization of health survey data might hinder the comparability of HLY estimates between countries (Brønnum-Hansen, 2014). Further, HLY can be estimated based on different statistical models using different health data (e.g., prevalence data of being unhealthy or incidence data of transitions between health states) and being healthy can be defined in various ways (e.g., being limited in daily activities or reporting a subjective health status), making HLY results sensitive to the choice of the calculation method. Thus, the challenge in obtaining comparable HLY estimates for different countries is harmonizing the estimation procedure in order to keep a potential bias due to differences in the methodology as small as possible (Jagger & Robine, 2011).

Another issue for cross-country comparison of HLY results, which has been less discussed so far, refers to population heterogeneity. Heterogeneity effects imply that members of a population do not all face the same health and mortality risks; therefore, a change in the population composition influences the HLY level of the total population (Luy et al., 2020; Guillot, 2011). This has been shown to be especially important for education and its relationship to mortality and health. Corresponding evidence indicates that besides changing mortality, a large proportion of improvements to longevity might be arise from the changing population structure according to education level (Hendi, 2017; Luy et al., 2019). Accordingly, education plays an important role in shaping health outcomes as differences in the population composition by educational attainment are highly relevant for assessing population health. Mathematically speaking, the HLY indicator can be seen as a population average, comprising several subpopulations with different health and mortality levels. Variations in HLY are therefore affected by differences in the population composition (i.e., the relative size of educational subpopulations in a given country) as well as by differences in the health and mortality levels (Vaupel & Canudas-Romo, 2002). In other words, a country's HLY value might be comparatively high (or low) due to a higher (or lower) share of highly educated individuals as opposed to disparities in the health and mortality levels of individuals.

In this paper, we derive gender-specific life tables on educational attainment for 16 European countries using Eurostat data. After combining the life tables with the prevalence of limitations in activities of daily living obtained from the EU-SILC survey, we provide estimates of $e_{x}$ and HLY based on women and men's educational attainment in 16 European countries. Further, we express total HLY as the sum of the education-specific HLYs weighted by the educational population structure. This allows us to investigate, whether differences in the population composition affect HLY estimates in addition to variations in health and mortality levels. We hypothesize that differences in HLY levels between countries can increase or decrease after adjusting for compositional effects. Finally, we discuss our results in the light of previous findings and summarize the main conclusions from this study.

## 2 Data and methods

### 2.1 Data

This analysis uses health and mortality data for European countries separated by age, gender, and educational attainment. Since educational institutions and qualifications are difficult to compare across countries, different approaches have been introduced to measure educational attainment (Schneider, 2010). In this analysis, we assess educational attainment according to the International Standard Classification of Education (ISCED). Individuals are defined as low educated when their highest level of attainment is lower secondary education or less (ISCED 0–2). A medium education level includes upper secondary or post-secondary non-tertiary education (ISCED 3–4). Those who attain tertiary education (ISCED 5–8) are considered highly educated. The ISCED is suitable for the purpose of this paper, since Eurostat relies on this framework and provides several statistics, including health and mortality data, for these ISCED groups.

In our paper, health status refers to the observed prevalence of any reported, long-lasting activity limitations of daily living, obtained from the European Union Statistics on Income and Living Conditions (EU-SILC). The Global Activity Limitation Indicator (GALI) defines individuals as healthy if they report no limitations at all. Using GALI is currently standard in EU policy and public health research (e.g., Jagger & Robine, 2011). For this reason, we choose calculating and reporting HLY estimates on the basis of GALI in this paper. GALI is based on the following question to determine such limitations:

For at least the past 6 months, to what extent have you been limited because of a health problem in activities people usually do? Would you say you have been (1) severely limited, (2) limited but not severely, or (3) not limited at all?

The indicator has been systematically assessed to obtain a harmonized health indicator, which enables researchers to compare the level of population health over time and across European countries (Jagger et al., 2010; Van Oyen et al., 2006). Yet, the challenges in obtaining comparable HLY estimates, which we have described in the introduction, still remain. Previous research indicates that some of the cross-country differences in population health measured through GALI may rather reflect differences in the implementation of GALI in the country-specific survey (e.g., wording of the question, data collection method, and the number of response categories) than actual health differentials between countries (Brønnum-Hansen, 2014; Berger et al., 2015, Rubio-Valverde et al., 2019). Accordingly, country differences in GALI should be interpreted with caution.

Table A1 provides information about the prevalence of being limited in daily activities and the sample sizes in EU-SILC 2016, which range from 2861 women and 2864 men in Sweden to 20,910 women and 18,985 men in Italy. As an example, the proportion of unhealthy Swedish men with a high education is about 6 percent, while the same proportion is about 20 percent in Slovakia (see table A1). We choose the year 2016 because it is the most recent year for which both, health and mortality is available.

Mortality data is usually provided in two dimensions, i.e., by age and sex, but not available for educational subpopulations separately. There are various ways to derive education-specific mortality estimates, which vary in feasibility (depending on the data collection system in the given country) and accuracy (depending on the quality of data in the given country). For this study, we used education-specific mortality data by age and gender taken from the Eurostat database,^1^ which are provided to Eurostat from national statistical institutes. The advantage of relying on this database is that it covers a large number of European countries and uses the same classification for educational groups as the EU-SILC, i.e., the ISCED. As a result, it allows estimating HLY by educational attainment for 16 European countries based on official statistics. The drawback, however, is that these 16 countries vary substantially in terms of the quality of their education-specific mortality data. While some countries use registers or census-linked death certificates, other estimates are based on unlinked death certificates or stem from survey samples (Corsini, 2010; Eurostat, 2015). These methodological differences can have a strong impact on the accuracy of education-specific mortality estimates and add further uncertainty to the HLY results presented in this study (e.g., Jasilionis et al., 2009). We evaluate the uncertainty in the education-specific mortality data provided by Eurostat in more detail in the supplementary material. In general, the mortality data for Nordic countries (Sweden, Norway, Finland, and Denmark) appear as being more reliable compared to Central and Eastern European countries. Due to the harmonization issues in both, health and mortality data, the findings presented in this study should not be treated as final results. They rather serve as a first empirical assessment, whether the population composition by educational attainment can have an impact on HLY levels in Europe.

### 2.2 Methods

#### 2.2.1 Deriving education-specific life tables from Eurostat

Eurostat does not provide complete period life tables by level of education, which are required to estimate education-specific HLY indicators. However, Eurostat publishes single age-specific estimates of remaining life expectancy by gender and educational attainment (as defined by the ISCED) for several European countries. We derived the missing life table function (i.e., the number of person-years lived between age *x* and *x* + *1*$\left(L_{x}\right)$) as the main life table function of interest for obtaining HLY based on the Sullivan method) from their single age-specific *e*_*x*_ estimates. Usually, *e*_*x*_ is the outcome of a complete life table. In this paper, we use *e*_*x*_ to reconstruct the (complete) life table, i.e., we calculated the life table in reverse. After assuming that in each age interval *x* to *x* + *1*, people dying within this period live an average 1/2 person-years ($a_{x}=0.5$), and using the life table function relationships (see Preston et al., 2001), we can express life table survivors *l*_*x+1*_ as:

(1) $$l_{x+1}=\frac{l_{x}\cdot \left(2\cdot e_{x}-1\right)}{1+2\cdot e_{x+1}}$$

where $l_{x}$ refers to the life table survivor at age x. Please note, $l_{0}$ denotes the life table radix (usually defined as 100,000) and does not require estimation. Thus, the Eurostat education-specific life tables can be recreated by an iterative process starting with $l_{1}$. Once all $l_{x}$ are estimated on the basis of equation (1), the remaining life table functions can be easily derived, such as $L_{x}$ ($L_{x}=\left(l_{x}+l_{x+1}\right)/2$). Theoretically, equation (1) enables us to reconstruct life table functions based on *e*_*x*_ values (under the *a*_*x*_ *=* 0.5 assumption). In practice, however, the reconstruction might require additional steps, which we describe in more detail in the supplementary material. In general, the proposed method leads to life tables, which produce accurate $e_{30}$ estimates, i.e., the difference between derived $e_{30}$ and original $e_{30}$ is mostly smaller than ±0.1 years. We focus on $e_{30}$ and HLY at age 30 as it is not only favorable from a technical point of view, but also theoretically: Very young persons have not usually finished their educational attainment (Connelly, Gayle, & Lambert, 2016).

#### 2.2.2 Estimating Healthy Life Years with the Sullivan method

The most commonly used approach for extending $e_{x}$ to HLY is the Sullivan method (Sullivan, 1971). It is based on the idea of applying the age-specific prevalence (proportions) of a population in an (un)healthy state to the age-specific person-years lived from the life table ($L_{x}\;\text{function}$). This enables us to divide the total life years for each age interval into those spent in good and poor health. Summing up only the *healthy* person-years lived across all ages yields HLY at age *x* by:

(2) $$HLY_{x}=\;\frac{1}{l_{x}}\;\sum_{i=x}^{\omega }{\;}_{n}\pi_{i}\;\cdot_{n}L_{i}\;,$$

with πin being the proportion of individuals in good health in the age interval *i* to *i + n* and Lin the number of person-years lived in the age interval *i* to *i + n*. The last open-age interval is represented by ω and refers to 85+ in our analyses.

The corresponding confidence intervals can be approximated by following the approach suggested by Jagger et al. (2014, p. 21). This method includes information about uncertainty arising from health survey data but ignores variance from the mortality data, which is usually justifiable^2^—and in our case, even inevitable—since we do not have information about the number of persons dying in each age interval. Hence, we only report confidence intervals for HLY, but not for $e_{x}$ and they reflect solely uncertainty due to random variation in the health survey data (not uncertainty in the mortality data).

#### 2.2.3 Expressing total HLY as the weighted sum of education-specific HLYs

As mentioned above, total HLY for a given population comprises the HLY contributions for several subpopulations. We follow the approach introduced by Shkolnikov et al. (2001) in order to decompose HLY at age *x* into the specific contribution of each subpopulation *i*. The aim of this method is dividing the overall period life table cohort into fractions corresponding to specific subpopulations, i.e., the low-, medium-, and high-educated subpopulations. Shkolnikov et al. (2001) use the fact that the sum of person-years lived by all subpopulations after age *x* must be equal to the total number of person-years lived by the whole cohort (Shkolnikov et al., 2001, p. 38). In the case of HLY, the period life table cohort includes only healthy person-years:

(3) $$healthy\;T_{x}=\overset{N}{\underset{i=1}{\sum }}healthy\;T_{x}^{i},$$

with $healthy\;{T^{i}}_{x}$being the number of healthy person-years lived from age *x* to the oldest age in subpopulation *i*. This leads to the problem of finding the period life table population weights $(\theta_{x}^{i}$) that satisfy this relationship, i.e., choosing $\theta_{x}^{i}$ in a way that group-specific healthy person-years sum up to the overall number of healthy person-years. In the case of three educational subpopulations, there are multiple solutions. For this reason, Shkolnikov et al. (2001) suggested including the group-specific proportions observed in the real population as additional constraints. We use the proportions of each educational subpopulation on the total population obtained from EU-SILC as constraints and estimated education-specific life table population weights by solving a system of linear equations. The exact estimation procedure with accompanying R code can be found in the supplementary material.

## 3 Results

### 3.1 Life expectancy and Healthy Life Years by educational attainment in 16 european countries

**Figure 1:**
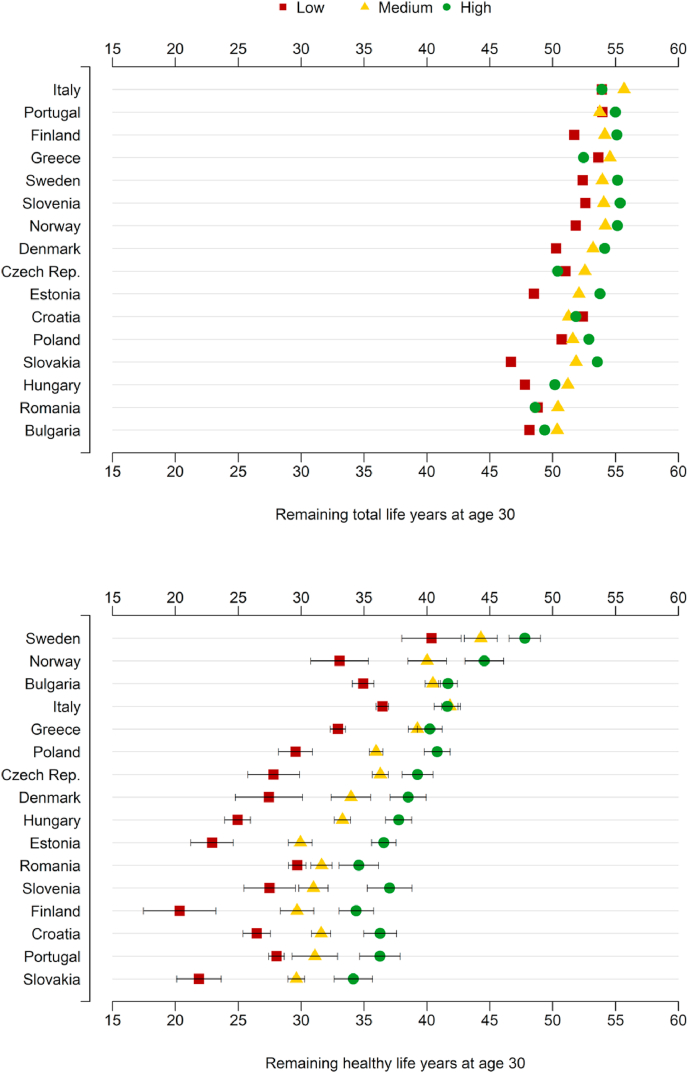
Life expectancy at age 30 ($e_{30}$) and Healthy Life Years at age 30 (HLY_30_) in 2016, with 95% confidence intervals for HLY for 16 European countries, by educational level, females. Source: Own calculations, using data from EU-SILC 2016 and Eurostat database. Notes: Countries ordered according to decreasing values in $e_{30}$ and HLY_30_. See Table A2 for the exact statistics. The reliability of $e_{30}$ and HLY_30_ estimates differs between countries due to differences in the quality of education-specific mortality data. See the supplement material for more details.

**Figure 2:**
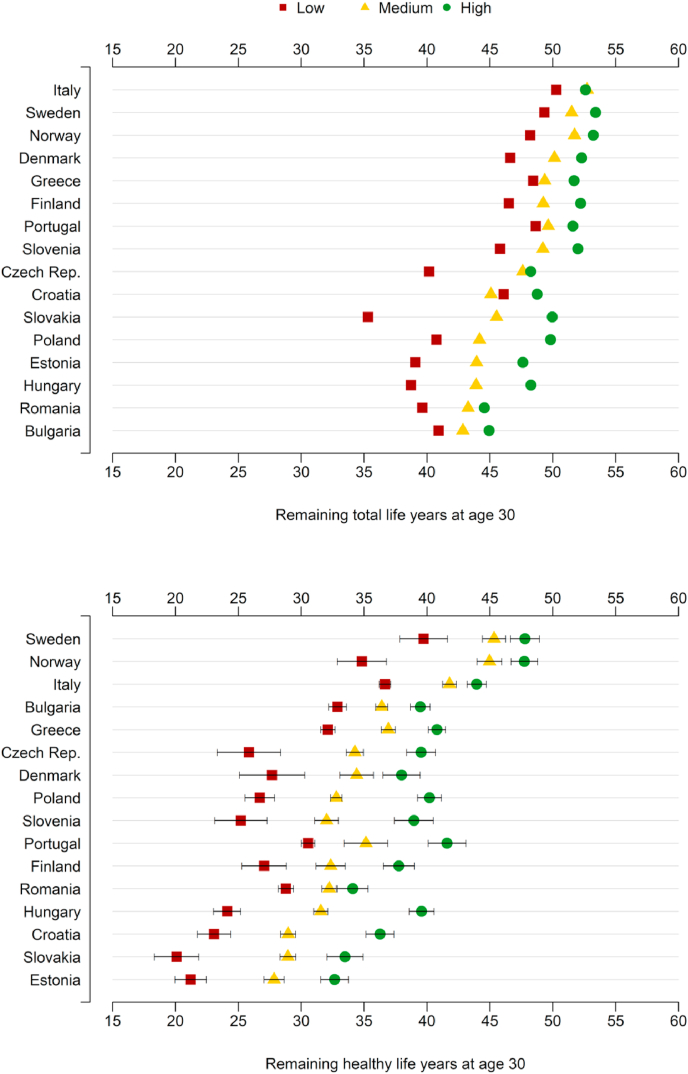
Life expectancy at age 30 ($e_{30}$) and Healthy Life Years at age 30 (HLY_30_) in 2016, with 95% confidence intervals for HLY for 16 European countries, by educational level, males. Source: Own calculations with data from EU-SILC 2016 and Eurostat database. Notes: Countries ordered according to decreasing values of $e_{30}$ and HLY_30_. See Table A3 for the exact statistics. The reliability of $e_{30}$ and HLY_30_ estimates differs between countries due to differences in the quality of education-specific mortality data. See the supplement material for more details.

Figure 1, Figure 2 show $e_{30}$ and HLY_30_ by educational attainment in 2016, stratified for sex. Three education-specific $e_{30}$ and HLY_30_ values are depicted for each country. In addition, 95% confidence intervals are included for HLY_30_ to reflect the uncertainty in the health data. The 16 countries are ordered by the country's $e_{30}$ and HLY_30_ rankings. Italy shows the highest $e_{30}$ among women and men, while Sweden is the top-ranked country in terms of HLY_30_ for both genders. Women and men in Bulgaria show the lowest observed $e_{30}$ level. The expected number of healthy life years measured through HLY_30_ is lowest in Slovakia (among women) and Estonia (among men). Further, educational inequalities in $e_{30}$ are largest in Slovakia (6.9 years for women and 14.7 years for men), while the difference between highly and low-educated Italians is relatively small (0.02 among women 2.32 among men). In general, educational inequalities are larger in HLY_30_ compared to $e_{30}$, ranging from 4.9 years in Romania (women) to 15.5 years in Hungary (men). For most countries included in this study, the differences between the educational subpopulations are statistically significant. The only exceptions are women in Bulgaria, Italy, and Greece, where estimates about highly and medium-educated individuals do not differ significantly.

Contradicting our expectations, the highly educated subpopulation does not always show the highest estimate in $e_{30}$ (i.e., in Italy, Greece, Czech Republic, Croatia, Hungary, Romania, and Bulgaria medium- or even low-educated individuals perform better than the highly educated subpopulation in terms of $e_{30}$). These findings can be most likely ascribed to the large uncertainty in the education-specific mortality data (see supplementary material). Since the HLY indicator is based on $e_{x}$ the issue of mortality data quality also affects the corresponding HLY estimates. As a result, country rankings and differences between countries in terms of $e_{30}$ and HLY and should be interpreted with caution. The imperfect harmonization of health data might relate to some of the unexpected results as well, e.g., Bulgaria ranks surprisingly high, while Denmark ranks considerably lower than other Nordic countries such as Sweden and Norway.

### 3.2 Decomposing total HLY_30_ in education-specific HLY_30_ estimates

As described in the methods section, total HLY can be expressed as the sum of the education-specific HLY estimates weighted by the population composition. Table 1 provides the corresponding results for the studied 16 European countries stratified for sex. Using the example of women in Bulgaria, medium-educated individuals make the greatest contribution to the total HLY_30_ level in 2016 (0.46 × 40.5/39.4 = 47%), while the contributions from highly and low-education persons are considerably lower (29.58% vs. 22.90%). In contrast, total HLY_30_ for women in Finland, Estonia, and Denmark largely arises from the contribution of highly educated individuals (about 50%). The difference between the Nordic countries and Bulgaria can be attributed to differences in the population composition, i.e., HLY_30_ for highly educated women is assigned to a much lower population weight in Bulgaria compared to the Nordic countries (0.28 vs. about 0.5). Thus, the decomposition demonstrates the importance of differences in the population structure for the HLY estimation. It adds useful information to the understanding of differences in HLY. For example, total HLY_30_ for men is slightly higher in Poland compared to Portugal (33.4 years vs. 32.3 years). Yet, all three education-specific HLY_30_ estimates are higher for men in Portugal, i.e., the low-, medium-, and highly educated men in Portugal can expect more healthy life years than their counterparts in Poland. Therefore, it is the population composition—a greater relative number of highly educated individuals—that leads to Poland's favorable performance in terms of HLY_30_. The same can be observed for women in Bulgaria and Italy. Comparing both populations according to education-specific HLY_30_ estimates indicates better population health in Italy. Again, the much higher share of low-educated individuals in Italy leads to a relatively higher total HLY_30_ value in Bulgaria (38.5 years vs. 39.4 years).

**Table 1:** Life table population weights (θi), HLY_30_ by education level, and contribution of educational groups on total HLY_30_ in 2016.

Educational composition (θi)				HLY_30_ by education				Contribution to total HLY_30_ (%)			
Country	High	Medium	Low	High	Medium	Low	Total	High	Medium	Low	Total
*Women*											
Bulgaria	0.28	0.46	0.26	41.7	40.5	34.9	39.4	29.6	47.5	22.9	100
Czech Rep.	0.2	0.68	0.12	39.3	36.3	27.8	35.9	22.2	68.7	9.1	100
Denmark	0.45	0.41	0.14	38.5	34.0	27.5	35.1	49.2	40.0	10.8	100
Estonia	0.45	0.4	0.15	36.6	29.9	22.9	31.9	51.9	37.3	10.8	100
Greece	0.15	0.31	0.54	40.2	39.3	32.9	36.0	16.6	34.1	49.3	100
Croatia	0.14	0.48	0.38	36.3	31.6	26.5	30.3	17.2	49.9	32.9	100
Italy	0.1	0.29	0.61	41.6	41.8	36.5	38.5	11.0	31.0	58.0	100
Hungary	0.22	0.52	0.26	37.8	33.3	25.0	32.1	25.9	54.2	19.9	100
Norway	0.38	0.38	0.24	44.6	40.0	33.1	40.1	42.3	37.8	19.9	100
Poland	0.25	0.56	0.19	40.8	36.0	29.6	35.9	28.2	55.8	16.0	100
Portugal	0.17	0.19	0.64	36.3	31.1	28.0	30.0	20.0	19.9	60.1	100
Romania	0.18	0.36	0.46	34.6	31.6	29.7	31.3	19.9	36.1	44.0	100
Slovenia	0.22	0.48	0.3	37.0	31.0	27.5	31.3	26.0	47.3	26.6	100
Slovakia	0.2	0.62	0.18	34.1	29.6	21.9	29.2	23.8	63.0	13.3	100
Finland	0.47	0.41	0.12	34.4	29.7	20.3	30.8	52.4	39.8	7.8	100
Sweden	0.28	0.4	0.31	47.8	44.3	40.4	44.0	30.7	40.4	28.9	100
*Men*											
Bulgaria	0.2	0.54	0.26	39.5	36.4	32.9	36.1	22.0	54.5	23.5	100
Czech Rep.	0.18	0.73	0.08	39.5	34.3	25.8	34.5	20.9	72.9	6.2	100
Denmark	0.34	0.46	0.2	38.0	34.4	27.7	34.3	37.5	46.5	16.0	100
Estonia	0.3	0.51	0.2	32.7	27.8	21.2	28.0	34.8	50.4	14.8	100
Greece	0.18	0.41	0.42	40.8	36.9	32.1	35.6	20.2	42.3	37.5	100
Croatia	0.17	0.63	0.2	36.3	29.0	23.1	29.0	20.9	63.1	16.0	100
Italy	0.09	0.36	0.55	44.0	41.8	36.7	39.1	9.6	38.8	51.6	100
Hungary	0.19	0.61	0.2	39.6	31.6	24.1	31.6	23.8	61.4	14.9	100
Norway	0.32	0.43	0.25	47.7	45.0	34.8	43.3	35.3	44.3	20.4	100
Poland	0.2	0.64	0.16	40.2	32.8	26.7	33.4	24.6	63.0	12.5	100
Portugal	0.07	0.22	0.71	41.6	35.1	30.6	32.3	8.4	24.1	67.5	100
Romania	0.24	0.47	0.29	34.1	32.2	28.8	31.7	26.0	48.1	25.9	100
Slovenia	0.22	0.61	0.17	39.0	32.0	25.2	32.4	26.4	60.1	13.6	100
Slovakia	0.18	0.71	0.11	33.5	28.9	20.1	28.8	21.2	71.4	7.4	100
Finland	0.24	0.48	0.28	37.8	32.3	27.1	32.2	28.4	48.0	23.7	100
Sweden	0.25	0.5	0.25	47.8	45.3	39.7	44.6	26.9	51.0	22.2	100

Source: Own calculations, using data from EU-SILC 2016 and Eurostat database.

One way to eliminate the effect of the population composition on HLY estimates is assuming a constant population composition by educational attainment for all analyzed countries. This standardization is presented in Table 2. Standardized HLY_30_ is estimated by assuming constant life table population weights (θi) for all 16 countries. We used the population composition by educational attainment of the EU-28 as the “reference” population.^3^ We refer to standardized HLY_30_ as education-adjusted HLY_30_ estimates. According to the education-adjusted HLY_30_ estimates, men in Portugal now show higher levels of population health compared to men in Poland (35.4 years vs. 33.0 years). Likewise, women in Italy show a higher number of healthy life years in terms of total HLY_30_ than women in Bulgaria after adjusting for differences in the population composition (education-adjusted HLY_30_ for Italian women is 40.2 years vs. 39.2 years for women in Bulgaria).

**Table 2:** Education-adjusted and original Healthy Life Years at age 30 for 16 European countries, 2016.

	Education-adjusted	Original	Change in	
Country	HLY_30_	HLY_30_	HLY_30_	Rank
*Women*				
Sweden	44.1	44.0	+0.0	1 → 1
Italy	40.2	38.5	+1.7	4 → 2
Norway	39.2	40.1	−0.9	2 → 3
Bulgaria	39.2	39.4	−0.2	3 → 4
Greece	37.7	36.0	+1.7	5 → 5
Poland	35.4	35.9	−0.6	6 → 6
Czech Rep.	34.6	35.9	−1.3	7 → 7
Denmark	33.3	35.1	−1.9	8 → 8
Hungary	32.0	32.1	−0.1	9 → 9
Romania	31.8	31.3	+0.6	11 → 10
Portugal	31.6	30.0	+1.6	15 → 11
Slovenia	31.5	31.3	+0.3	12 → 12
Croatia	31.3	30.3	+1.0	14 → 13
Estonia	29.6	31.9	−2.3	10 → 14
Slovakia	28.6	29.2	−0.6	16 → 15
Finland	28.2	30.8	−2.6	13 → 16
*Men*				
Sweden	44.3	44.6	−0.2	1 → 1
Norway	42.8	43.3	−0.5	2 → 2
Italy	40.9	39.1	+1.7	3 → 3
Greece	36.5	35.6	+0.9	5 → 4
Bulgaria	36.2	36.1	+0.1	4 → 5
Portugal	35.5	32.3	+3.2	10 → 6
Denmark	33.4	34.3	−0.9	7 → 7
Czech Rep.	33.2	34.5	−1.3	6 → 8
Poland	33.0	33.4	−0.4	8 → 9
Finland	32.2	32.2	+0.1	11 → 10
Slovenia	31.8	32.4	−0.5	9 → 11
Romania	31.7	31.7	+0.0	12 → 12
Hungary	31.5	31.6	−0.1	13 → 13
Croatia	29.2	29.0	+0.2	14 → 14
Slovakia	27.6	28.8	−1.3	15 → 15
Estonia	27.2	28.0	−0.8	16 → 16

Source: EU-SILC 2016 and Eurostat database (own calculations).

Notes: The population composition by educational attainment for the EU-28 serves as the “reference” population.

The exact life table population weights are 0.29 (low-educated), 0.45 (medium-educated), and 0.26 (highly educated).

## 4 Discussion

In this article, we investigated the role of education in assessing population health across Europe according to the HLY indicator. While previous studies have mainly focused on issues connected to the imperfect harmonization of health survey data, we addressed how population composition impacts HLY estimation. As expected, we observed large educational inequalities in HLY_30_, which substantially exceed inequalities in $e_{30}$. The greatest gap between low- and highly educated individuals was found among men in Hungary. While persons with low education can expect 24.1 HLY_30_, HLY_30_ for highly educated individuals is almost 40 years. Moreover, European countries differ considerably with respect to their population composition by educational attainment. For example, the population of low-educated women in Portugal is about 62 percent as opposed to about 20 percent in Poland. This points to the importance of differences in population composition for assessing population health.

We expressed each total HLY_30_ as the sum of education-specific HLY_30_ and weighted by the size of education-specific subpopulations to demonstrate how educational attainment affects population composition. For example, total HLY_30_ among men is higher in Poland than in Portugal (33.4 years vs. 32.3 years). However, looking at education-specific HLY_30_ values suggests that Portuguese men expect to live healthier lives than Polish men in all three educational subpopulations. It is, therefore, the larger number of low-educated individuals in Portugal that drives the comparatively low total HLY_30_ value. In this sense, a comparison of total HLY_30_ between Portugal and Poland reflects more differences in the population composition by educational attainment as opposed to inequality in people's health and mortality levels. Controlling for the effect of the population composition by education level on HLY_30_ by means of standardization leads to a higher HLY_30_ value in Portugal compared to Poland. Thus, researchers and policy makers should be aware of the fact that differences in HLY across Europe are not only driven by disparities in the health and mortality levels between countries, but also influenced by differences in the population composition by education level.

The relationship between education and population health is well documented in the literature. Luy et al. (2019) have shown that the improvements in $e_{x}$ between 1990 and 2010 in Italy, Denmark, and the USA partly arose from an increasing proportion of higher educated individuals. In addition, Deboosere et al. (2009) pointed to the importance of considering shifts in the population composition according to educational attainment in their analysis of how $e_{x}$ progressed in Belgium from 1991 to 2014. Likewise, Shkolnikov et al. (2006) emphasized how changes in the educational population structure played a role in mortality trends in Central and Eastern-Europe during the 1990s. Our findings suggest that changes in the population composition according to educational attainment might affect HLY trends even more than $e_{x}$ trends, because differences by education level are larger in HLY compared to $e_{x}$.

The impact of heterogeneity on population averages has been studied previously. It has been shown, for example, that the mortality patterns of specific subpopulations can considerably differ from the mortality pattern experienced by the aggregated population (Vaupel & Yashin, 1985). It is even possible that the life expectancy for the overall population lies outside the range of its subpopulations (Andreev et al., 1989). The presented results in our study relate to this discussion in the sense that they demonstrate population averages cannot be used to infer experiences from subpopulations. It is important to note, however, that this does not limit the appropriateness of using population averages for studying differences in HLY between countries. Knowledge about levels and trends in HLY for the aggregated population is without a doubt worthwhile. The advantage of examining specific subpopulations (e.g., by education, income or geography) and adjusting for compositional effects is adding further information, which allows a deeper understanding of differences in HLY.

This knowledge can be valuable for policy makers in order to introduce health interventions more targeted. While some countries can increase total HLY by reducing inequalities and promoting education, others need to target structural disadvantages, e.g., basic conditions for establishing population health such as a well-functioning healthcare system. The relatively large number of HLY for highly educated men in Portugal suggests a great potential for improving total HLY through educational expansion, which could even have implications for population forecasting in health and mortality. In contrast, Romania shows similar levels of HLY_30_ for all three educational groups, indicating that other factors (e.g., structural problems in the healthcare system that concerns all educational groups) may prevent Romanians from living long and healthy lives regardless of their educational attainment.

The methodological limitations of this study should be mentioned. We presented HLY as a weighted average of education-specific HLYs by deriving life table population weights. Our method assumes a constant population composition over ages, i.e., all ages show the same composition by educational attainment. A more sophisticated method^4^ might improve the accuracy of the decomposition but will most likely not change the overall conclusion drawn from this study, i.e., the HLY indicator is strongly associated with the level of education and consequently, differences in the population's educational composition affect HLY levels. Yet, some of the analyzed countries are more (or less) affected and due to the methodological limitations, it is difficult to pinpoint the reasons for the observed cross-country differences.

First, we noted that the imperfect harmonization of health data affects how educational differences in HLY can be compared across Europe (Rubio-Valverde et al., 2019). We observe that Sweden and Norway show considerable higher HLY_30_ values compared to the other analyzed European countries, which is likely to reflect more differences in the implementation of GALI in the country-specific health surveys than differences in actual health and mortality levels (Brønnum-Hansen, 2014). Methodological differences such as formulation of a question or how the GALI question is filtered can lead to a comparatively high/low prevalence of being limited in daily activities. Second, the derived weights for our decomposition are based on the proportions of each educational subpopulation on the total population obtained from EU-SILC. National health surveys, however, do not always accurately reflect the population composition by educational attainment in a given country (Spitzer, 2020). This can affect the estimation of education-specific life table population weights and potentially bias our results. Third, the history of the educational expansion in a given country is likely to have an impact on the presented estimates. Italy, for example, started its educational transition comparatively late and a large proportion of older cohorts show a low educational level (Luy et al., 2019). Younger Italians attained higher levels of education and their share might be underestimated in the presented results (i.e., the exceptionally low population weights for high-educated individuals in Italy shown in Table 1 might underestimate their actual contribution to HLY).

Another methodological complexity refers to examining the impact of education on HLY on the basis of a synthetic-cohort approach, i.e., using the period life table population as a model for studying actual groups of individuals. In terms of HLY, the period life table links together the age-specific health and mortality information from individuals with substantially different set of historical conditions and behaviors (Guillot & Canudas-Romo, 2006; Vaupel et al., 1979; Luy et al., 2020). From a conceptual point of view, it is therefore more reasonable to study health and mortality from a cohort perspective (Guillot, 2011; Sauerberg et al., 2020). This holds in particular for the case of education because educational expansion is mostly a cohort-based phenomenon and its progress differs between countries. As an example, the share of high (or low) educated individuals at certain ages can differ considerably between countries and consequently mortality selection might play a role for cross-country comparisons based on a synthetic cohort approach. Unfortunately, data restrictions usually hamper the feasibility of analyzing education-specific health and mortality levels from the theoretically favored cohort perspective.

Furthermore, the education-specific mortality data provided by Eurostat is only available up to age 85+ and, therefore, the last open-age interval in our derived life tables starts inevitable at age 85. In other words, we assume a constant hazard of death for individuals being 85 years or older. Missov et al. (2016) have demonstrated how starting a constant-hazard assumption at a relatively young age can lead to erroneous conclusions about the populations's $e_{x}$ level. This has important implications for our results because we analyze educational subpopulations in low-mortality countries. Especially for medium- and high-educated women, an open-age interval at an older age (e.g., 100+) would be more appropriate. With regard to HLY, this would also require available health data for the oldest old. The EU-SILC, however, provides health data only up to age 85+. Additionally, survey samples such as the EU-SILC do not include the institutionalized population which is likely to limit the accuracy of prevalence data for older individuals (Kelfve et al., 2013). The sensitivity analysis shown in the supplement indicates HLY_30_ varies by about one year depending on the choice of the last open-age interval. Finally, we have emphasized the harmonization issues in both, health and mortality data in the data section and recommended interpreting the presented results with caution.

## 5 Conclusions

Despite the aforementioned limitations, our analysis provides important insights on the measurement of population health across Europe using the HLY indicator. First, we find large educational inequalities in HLY and education-specific HLY values differs considerably from the total HLY value. This finding highlights that total HLY should not be used to infer health and mortality levels for specific population groups. Second, the decomposition reveals the important relationship between the population composition by educational attainment and HLY. We examined the impact of compositional effects on HLY by means of standardization. The standardized (or education-adjusted) HLY estimates do not differ much from conventional HLY estimates, suggesting a rather small impact of the population composition by educational attainment on HLY levels for the 16 European countries included in this study. The case of Portugal, however, serves as an empirical example that compositional effects can indeed play an important role for assessing population health on the basis of HLY. After adjusting for this effect, Portugal's HLY level increased considerably (from rank 15 to 11 for women and from rank 10 to 6 for men). We, therefore, conclude education-adjusted HLY might be more important in comparing countries that experienced larger differences in their educational expansion. Adjusting for compositional effects can then add useful information to the understanding of variations in HLY across countries, i.e., it reveals whether those differences stem from group-specific health differentials or from differences in the population structure.

## Funding

This work has received funding from the European Research Council under the EU’s Horizon 2020 Research and Innovation Programme, Grant Agreement No. 725187 (LETHE).

## Author statement

Markus Sauerberg designed the study, carried out the analyses and wrote the paper.

## Ethics approval

Not required.

## Submission declaration

An older version of the manuscript has been published as a working paper^1^ and was part of Markus Sauerberg's PhD thesis submitted to the Vienna University of Economics and Business (WU Vienna). The current version of the manuscript is not under review for publication elsewhere.

## Declaration of competing interest

The authors declare that they have no known competing financial interests or personal relationships that could have appeared to influence the work reported in this paper.

## Figures and Tables

**Table A1:** Limitations in activities of daily living (GALI) by education level and educational distribution, by country, in 2016

			Activity limitations (%)			Educational distribution (%)		
Country		*N*	Low	Medium	High	Low	Medium	High
Bulgaria	Women	8257	29.61	15.59	10.59	29.68	44.87	25.45
	Men	7258	20.42	11.75	9.87	27.68	53.99	18.32
Czech Rep.	Women	6924	44.99	26.04	13.21	14.90	67.11	17.99
	Men	4590	28.63	25.00	14.43	7.98	73.49	18.54
Denmark	Women	3296	40.37	34.44	25.49	23.99	40.20	35.80
	Men	2956	34.26	29.11	24.19	23.04	45.73	31.23
Estonia	Women	6630	52.21	35.68	24.84	18.46	39.69	41.85
	Men	5805	40.44	28.51	24.92	22.07	50.40	27.52
Greece	Women	19,581	46.00	15.29	11.21	40.31	36.62	23.08
	Men	18,269	38.71	14.49	13.35	34.66	41.30	24.04
Croatia	Women	8869	57.00	25.13	17.47	34.12	48.00	17.88
	Men	7935	47.09	26.51	18.83	21.03	63.28	15.69
Italy	Women	20,910	36.87	14.51	12.70	49.25	34.91	15.84
	Men	18,985	27.35	12.24	10.68	47.26	38.21	14.54
Hungary	Women	8718	48.83	27.27	16.12	25.96	52.22	21.82
	Men	7045	32.42	22.90	13.15	18.96	61.48	19.56
Norway	Women	3313	31.43	24.03	14.70	29.24	37.18	33.58
	Men	3465	20.93	11.55	7.84	28.30	41.74	29.96
Poland	Women	13,294	43.29	23.83	11.59	19.50	55.72	24.78
	Men	10,941	30.53	21.03	11.03	16.52	64.11	19.38
Portugal	Women	12,148	48.43	21.00	18.84	61.83	18.74	19.43
	Men	10,532	34.95	16.07	13.78	64.28	21.27	14.44
Romania	Women	8308	39.53	24.83	15.36	50.62	36.29	13.09
	Men	7487	25.04	19.29	13.97	40.55	45.16	14.29
Slovenia	Women	4489	56.25	37.36	24.80	24.83	46.69	28.48
	Men	4096	41.96	29.03	20.62	15.69	60.68	23.62
Slovakia	Women	7498	53.54	32.22	16.76	16.87	62.12	21.01
	Men	6366	30.41	26.68	20.58	11.32	71.03	17.64
Finland	Women	5184	58.67	38.51	30.84	25.20	38.62	36.18
	Men	5363	45.12	26.94	23.21	22.98	47.64	29.37
Sweden	Women	2861	23.68	15.46	11.38	26.24	40.41	33.36
	Men	2864	18.81	10.55	6.48	22.55	50.65	26.80
								

Source: EU-SILC 2016 (own calculations).

**Table A2:** Life expectancy at age 30 ($e_{30}$) and Healthy Life Years at age 30 (HLY_30_) by educational level in 2016, with 95% confidence intervals for 16 European countries, females

	$e_{30}$						HLY_30_ with 95% confidence intervals				
Country	Low		Medium	High		Total	Low		Medium	High	Total
Bulgaria	48.15		50.36	49.36		49.85	34.93 (34.07–35.79)		40.47 (39.88–41.07)	41.68 (40.93–42.43)	39.38 (38.99–39.77)
Czech Rep.	51.03		52.56	50.40		52.42	27.81 (25.76–29.86)		36.30 (35.67–36.93)	39.26 (38.03–40.49)	35.90 (35.38–36.42)
Denmark	50.28		53.21	54.14		52.82	27.45 (24.77–30.12)		33.96 (32.39–35.54)	38.51 (37.08–39.94)	35.10 (34.22–35.98)
Estonia	48.51		52.09	53.77		52.41	22.92 (21.24–24.60)		29.94 (28.99–30.89)	36.57 (35.60–37.55)	31.90 (31.31–32.48)
Greece	53.63		54.55	52.45		54.03	32.93 (32.32–33.53)		39.25 (38.53–39.96)	40.23 (39.24–41.21)	35.99 (35.68–36.30)
Croatia	52.40		51.26	51.85		52.11	26.46 (25.38–27.55)		31.59 (30.83–32.36)	36.28 (34.98–37.59)	30.33 (29.86–30.81)
Italy	53.90		55.67	53.92		54.71	36.46 (35.97–36.96)		41.83 (41.18–42.48)	41.63 (40.59–42.66)	38.52 (38.22–38.83)
Hungary	47.79		51.21	50.18		50.28	24.95 (23.92–25.98)		33.29 (32.63–33.94)	37.76 (36.72–38.79)	32.13 (31.68–32.59)
Norway	51.85		54.19	55.15		53.93	33.05 (30.76–35.35)		40.02 (38.49–41.55)	44.56 (43.02–46.10)	40.07 (39.20–40.94)
Poland	50.72		51.60	52.88		52.00	29.55 (28.21–30.90)		35.96 (35.42–36.50)	40.82 (39.80–41.84)	35.92 (35.53–36.30)
Portugal	53.95		53.74	55.00		54.15	28.04 (27.43–28.65)		31.10 (29.29–32.90)	36.26 (34.65–37.88)	29.99 (29.53–30.44)
Romania	48.79		50.42	48.61		50.09	29.70 (29.01–30.39)		31.63 (30.78–32.48)	34.58 (33.01–36.16)	31.27 (30.80–31.74)
Slovenia	52.61		54.06	55.37		53.95	27.50 (25.46–29.53)		30.98 (29.82–32.14)	37.04 (35.26–38.81)	31.26 (30.50–32.02)
Slovakia	46.68		51.86	53.55		51.49	21.88 (20.11–23.64)		29.62 (28.95–30.28)	34.14 (32.61–35.67)	29.16 (28.65–29.68)
Finland	51.72		54.14	55.10		54.06	20.34 (17.46–23.23)		29.67 (28.34–31.01)	34.38 (33.00–35.76)	30.78 (30.04–31.53)
Sweden	52.39		53.94	55.17		53.97	40.36 (38.01–42.72)		44.29 (42.97–45.61)	47.78 (46.53–49.03)	44.04 (43.26–44.83)
						

Note: The reliability of $e_{30}$ and HLY_30_ estimates differs between countries due to differences in the quality of education-specific mortality data.

See the supplement material for more details.

**Table A3:** Life expectancy at age 30 ($e_{30}$) and Healthy Life Years at age 30 (HLY_30_) by educational level in 2016, with 95% confidence intervals for 16 European countries, males

	$e_{30}$				HLE_30_ with 95% confidence intervals			
Country	Low	Medium	High	Total	Low	Medium	High	Total
Bulgaria	40.93	42.85	44.94	43.08	32.89 (32.18–33.60)	36.40 (35.92–36.88)	39.47 (38.69–40.25)	36.11 (35.76–36.46)
Czech Rep.	40.18	47.61	48.25	47.27	25.84 (23.32–28.37)	34.27 (33.59–34.94)	39.53 (38.38–40.67)	34.53 (33.95–35.10)
Denmark	46.62	50.13	52.29	49.89	27.68 (25.09–30.28)	34.41 (33.07–35.75)	37.98 (36.50–39.45)	34.28 (33.39–35.17)
Estonia	39.06	43.94	47.62	44.47	21.21 (19.96–22.46)	27.84 (27.04–28.64)	32.65 (31.56–33.75)	27.98 (27.43–28.54)
Greece	48.44	49.36	51.70	49.51	32.12 (31.55–32.69)	36.92 (36.36–37.48)	40.79 (40.11–41.47)	35.61 (35.32–35.90)
Croatia	46.10	45.08	48.76	46.35	23.07 (21.74–24.39)	28.96 (28.37–29.55)	36.26 (35.16–37.37)	29.00 (28.53–29.47)
Italy	50.28	52.73	52.60	51.48	36.66 (36.20–37.11)	41.79 (41.24–42.33)	43.95 (43.19–44.70)	39.14 (38.85–39.44)
Hungary	38.72	43.91	48.26	43.75	24.11 (23.03–25.19)	31.55 (31.00–32.11)	39.57 (38.58–40.56)	31.62 (31.18–32.06)
Norway	48.21	51.73	53.22	51.42	34.83 (32.88–36.78)	44.96 (43.98–45.94)	47.74 (46.68–48.80)	43.28 (42.59–43.98)
Poland	40.75	44.17	49.82	45.03	26.71 (25.54–27.88)	32.79 (32.34–33.24)	40.20 (39.25–41.15)	33.35 (32.99–33.72)
Portugal	48.65	49.63	51.60	49.15	30.55 (30.01–31.08)	35.14 (33.41–36.88)	41.60 (40.10–43.10)	32.28 (31.83–32.73)
Romania	39.63	43.27	44.56	43.25	28.78 (28.19–29.38)	32.23 (31.65–32.81)	34.10 (32.88–35.31)	31.70 (31.30–32.09)
Slovenia	45.81	49.21	51.99	49.04	25.21 (23.13–27.29)	32.01 (31.07–32.96)	38.96 (37.41–40.51)	32.35 (31.63–33.08)
Slovakia	35.31	45.53	49.96	45.19	20.09 (18.33–21.85)	28.94 (28.32–29.56)	33.49 (32.05–34.92)	28.83 (28.30–29.35)
Finland	46.51	49.24	52.22	49.48	27.05 (25.28–28.83)	32.34 (31.18–33.51)	37.76 (36.53–38.99)	32.16 (31.49–32.83)
Sweden	49.33	51.50	53.40	51.45	39.73 (37.83–41.63)	45.33 (44.41–46.25)	47.79 (46.63–48.94)	44.55 (43.91–45.20)

Note: The reliability of $e_{30}$ and HLY_30_ estimates differs between countries due to differences in the quality of education-specific mortality data.

See the supplement material for more details.

## Footnotes

1 https://ec.europa.eu/eurostat/data/database. A description of the metadata is given at https://ec.europa.eu/eurostat/cache/metadata/en/demo_mor_esms.htm.

2 Death rates are usually calculated from administrative data and not derived from survey samples. In this case, we do not have any information about death counts and exposure as we derived our life tables from *e*_*x*_ (see section 2.2.1).

3 The exact life table population weights are 0.29 (low-educated), 0.45 (medium-educated), and 0.26 (high-educated). For example, the education-adjusted HLY_30_ for women in Bulgaria is calculated as: 41.68⋅ 0.26 + 40.47 ⋅0.45 + 34.93 ⋅0.29 = 39.18.

4 One option would be modifying the approach introduced by Torres et al. (2019). This would, however, require calculating HLY based on transition rates between health states in a multistate framework (see supplement for more details).
