# Supplementary material for: The impact of population's educational composition on Healthy Life Years: An empirical illustration of 16 European countries
Source: SSM Popul Health. 2021 Jun 26;15:100857. doi: 10.1016/j.ssmph.2021.100857 (PMC8255240; doi:10.1016/j.ssmph.2021.100857)
Supplement: Multimedia component 1 [file mmc1.pdf]

# Supplementary information

## Deriving education-specific life tables from Eurostat

The life table survivors at age $x$ ($l_x$) can be obtained from life expectancy estimates at age $x$ ($e_x$) after assuming that in each age interval $x$ to $x+1$, people dying within this period live on average 1/2 person-years ($a_x = 0.5$):

$$l_{x+1} = \frac{l_x \cdot (2 \cdot e_x - 1)}{1 + 2 \cdot e_{x+1}}. \tag{1}$$

Please note, $l_0$ denotes the life table radix (usually defined as 100 000) and does not require estimation. Thus, the life table reconstruction starts with deriving $l_1$:

$$l_1 = \frac{l_0 \cdot (2 \cdot e_0 - 1)}{1 + 2 \cdot e_1}. \tag{2}$$

In this way, the life table survivors at age 1 can be estimated from three known life table functions, i.e., $l_0$, $e_0$, and $e_1$. In the next step, $l_2$ is estimated from $l_1$, $e_1$, and $e_2$ and so forth. Once all $l_x$ are estimated on the basis of this algorithm, the remaining life table functions can be easily derived, such as $L_x$ ($L_x = (l_x + l_{x+1})/2$). Theoretically, equation 1 enables us to reconstruct life table functions based on $e_x$ values (under the $a_x = 0.5$ assumption). In practice, however, the reconstruction might require additional steps. For example, the $e_x$ values provided by Eurostat have only one decimal place. This limits the accuracy of the $l_x$ derivation and might result in constant $l_x$ values for several ages. To overcome this issue, we fitted a non-parametric curve to the data and predicted $e_x$ values with more decimal places. More specifically, we used the loess() function in R in order to obtain $e_x$ values with more decimal places that are as close as possible to the original $e_x$ values. In some cases, e.g., for the highly educated subpopulation in very low-mortality countries, the proposed derivation procedure still produces constant $l_x$ values at young ages. We solved this issue by focusing on $e_{30}$ and HLY at age 30.

The following code provides an example for calculating education-specific life tables when only the education-specific $e_x$ values are known. In other words, the aim of the code is to calculate the life table backwards, namely from $e_x$ to $p_x$. This is necessary because Eurostat does not provide education-specific life tables, but education-specific $e_x$ values are available. Please note, the results in this example will differ from the results in the paper due to updates in the Eurostat database.

```r
library(dplyr)
library(eurostat)
#please load these packages and download the data like this:
data <- get_eurostat("demo_mlexpecedu", time_format = "num")

#rename and redefine the file
data$isced11 <- as.character(data$isced11)
data$isced11 <- ifelse(data$isced11=="ED0-2", "lower", data$isced11)
data$isced11 <- ifelse(data$isced11=="ED3_4", "middle", data$isced11)
data$isced11 <- ifelse(data$isced11=="ED5-8", "higher", data$isced11)
data$isced11 <- ifelse(data$isced11=="TOTAL", "total", data$isced11)

data$age <- as.character(data$age)
data$age <- ifelse(data$age=="Y_LT1", "Y0", data$age)
data$age <- ifelse(data$age=="Y_GE85", "Y85", data$age)
data$age <- substring(data$age, 2)
```

```r
data <- data[,-1]
colnames(data) <- c("sex","age","edu","country","year","ex")
data$age <- as.numeric(data$age)
#Filter for the year 2016, as we have done
data <- filter(data, year==2016)
```

The following function has the arguments “country.select”, “edu.select” and “sex.select”. Thus, the funcation allows to derive life tables for each educational level (high, middle, low, and total), for each country with available data (16 European countries), separated for men and women.

```r
my.function <- function(country.select, edu.select, sex.select) {

    select.country <- arrange(filter(data, country==country.select ,edu==edu.select &
                                              sex==sex.select),age)

#smooth to get more decimals by applying the loess function,
#then predict ex with more decimals
    grab.LE <- select.country$ex
    smooth.it <- loess(grab.LE~select.country$age, span=0.2)
    predict.it <- predict(smooth.it, seq(0,85,1))
    select.country$ex.decimals <- predict.it

    LT.derive <- data.frame(Age=0:85)
    LT.derive$lx <- NA

    LT.derive$ex <- select.country$ex.decimals
    LT.derive$lx[1] <- 100000
    LT.derive$Tx[1] <- 100000*select.country$ex.decimals[1]

#this loop refers to equation 1 in the paper
    for (j in 2:86) {

        upper <- LT.derive$lx[j-1]*(2*LT.derive$ex[j-1]-1)
        bottom <- 1+2*LT.derive$ex[j]
        LT.derive$lx[j] <- upper/bottom
    }
#Checks if lx is monotonic decreasing, i.e., no resurrection in the life table
    lx.diff <- diff(LT.derive$lx)
    lx.diff <- round(lx.diff, 5)

    if (all(diff(lx.diff) < 0)) {

        px <- c(LT.frame$lx[-1]/LT.frame$lx[-86],0)

    }else{
#sometimes, it is not, so I force it =)
#please note, this occurs usually at very young ages and won't affect
#LE at age 30 or older
        lx.diff[lx.diff>=0] <- -runif(length(lx.diff[lx.diff>=0]), 1, 5)
        lx.monotonic <- cumsum(c(100000, lx.diff))
        px <- c(lx.monotonic[-1]/lx.monotonic[-86],0)

        }
#from here, the life table is constructed very standardly
```

```
    lx <- round(c(100000, (cumprod(px)*100000)[1:(length(px)-1)]))
    dx <- round(c(-diff(lx), lx[length(lx)]))
    LT.derive$lx <- lx
    LT.derive$dx <- dx
    LT.derive$px <- px
    Lx1 <- lx[-1]+0.5[-length(px)]*dx[-length(dx)]
    Lx.open <- LT.derive$Tx[1]-sum(Lx1)
    LT.derive$Lx <- round(c(Lx1, Lx.open))
    LT.derive$Tx <- rev(cumsum(rev(LT.derive$Lx)))
    LT.derive$ex.derived <- LT.derive$Tx/LT.derive$lx
    LT.derive$ex.original <- select.country$ex
    LT.derive$diff <- LT.derive$ex.original-LT.derive$ex.derived
    LT.derive$Country <- country.select
    LT.derive$Edu <- edu.select
    LT.derive$Sex <- sex.select

    return(LT.derive[,c("Country","Edu","Sex","Age","px","lx","dx","Lx",
                        "Tx","ex.derived","ex.original","diff")])
}
```

The following code applies the function to all 16 European countries by educational attainment, stratified by sex.

```
#these are the country codes
edu.countries <- c("BG","DK","EE","EL","HR","IT","HU", #CZ is currently not available
                   "PL","PT","RO","SI","SK","FI","SE","NO")


###Females###
out.females <- c()

for (country.select in edu.countries) {

    for (edu.select in c("higher","middle","lower")) {

        out.females <- rbind(out.females,my.function(country.select, edu.select, "F"))
}
}


###Males###
out.males <- c()

for (country.select in edu.countries) {

    for (edu.select in c("higher","middle","lower")) {

        out.males <- rbind(out.males,my.function(country.select, edu.select, "M"))
}
}
```

Finally, I plot the difference between the original $e_x$ and the derived $e_x$.

```
par(mfrow=c(3,3))
for (edu in c("higher","middle","lower")) {
```

```
    plot(1,1, type="n", xlim=c(1,16), ylim=c(-0.2,0.2),
         main=paste("Females",edu,sep=" "), xlab="Countries",
         ylab="LE 30 original - LE30 derived")
    points(1:15,out.females$diff[out.females$Edu==edu & out.females$Age==30])
    text(1:15,out.females$diff[out.females$Edu==edu & out.females$Age==30], 1:16,
         label=out.females$Country[out.females$Edu==edu & out.females$Age==30])
}

for (edu in c("higher","middle","lower")) {
    plot(1,1, type="n", xlim=c(1,16), ylim=c(-0.2,0.2),
         main=paste("Males",edu,sep=" "), xlab="Countries",
         ylab="LE 30 original - LE30 derived")
    points(1:15,out.males$diff[out.males$Edu==edu & out.males$Age==30])
    text(1:15,out.males$diff[out.males$Edu==edu & out.males$Age==30], 1:16,
         label=out.males$Country[out.males$Edu==edu & out.males$Age==30])
}
```

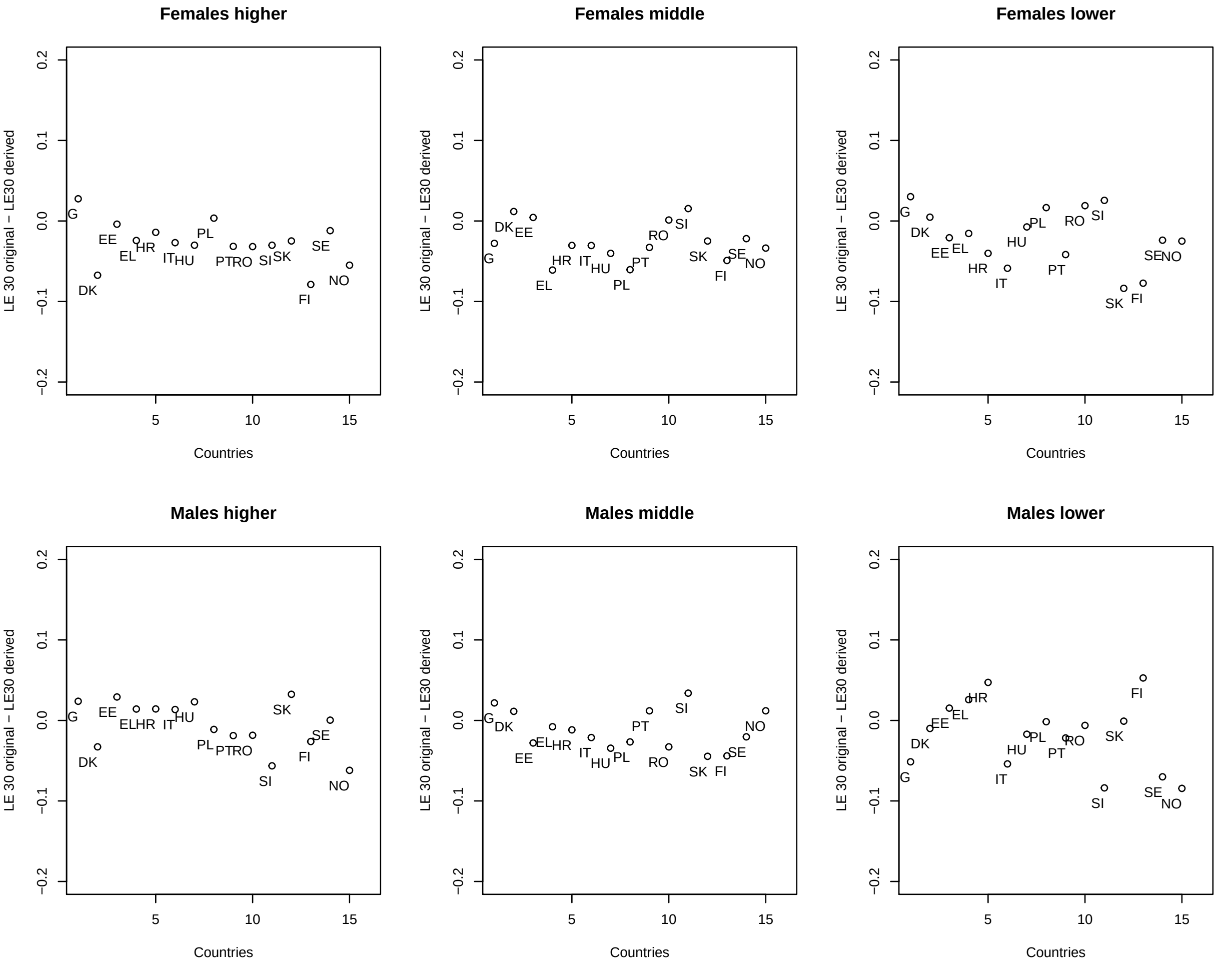
Females higher
Females middle
Females lower
Males higher
Males middle
Males lower
LE 30 original − LE30 derived
Countries
G
DK
EE
EL
HR
IT
HU
PL
PT
RO
SI
SK
SE
FI
NO

### Complete life tables by age and education (stratified by women and men)

This prints all the age- and education-specific life tables (the output it omitted).

```
library(knitr)

table.fun <- function(country.select) {

    print(
        kable(filter(out.females, Country==country.select & Edu=="higher"),
              digits=4, caption=paste("Life table for high-educated women in",
                                      country.select,", 2016",sep=" "))
        )
    print(
        kable(filter(out.females, Country==country.select & Edu=="middle"),
              digits=4, caption=paste("Life table for middle-educated women in",
                                      country.select,", 2016",sep=" "))
          )
    print(
        kable(filter(out.females, Country==country.select & Edu=="lower"),
              digits=4, caption=paste("Life table for low-educated women in",
                                      country.select,", 2016",sep=" "))
        )

    print(
        kable(filter(out.males, Country==country.select & Edu=="higher"),
              digits=4, caption=paste("Life table for high-educated men in",
                                      country.select,", 2016",sep=" "))
            )

    print(
        kable(filter(out.males, Country==country.select & Edu=="middle"),
              digits=4, caption=paste("Life table for middle-educated men in",
                                      country.select,", 2016",sep=" "))
            )
    print(
        kable(filter(out.males, Country==country.select & Edu=="lower"),
              digits=4, caption=paste("Life table for low-educated men in",
                                      country.select,", 2016",sep=" "))
            )
}

for (country in edu.countries) {
    table.fun(country)
}
```

## Decomposing healthy life expectancy into group-specific healthy life expectancies

Period life expectancy at age $x$ is usually expressed in terms of the period survivorship function ($l_x$)

$$e_x = \frac{1}{l_x}\int_x^{\infty} l_a da, \quad (1)$$

where $l_x$ defines the probability of surviving from birth to age $x$. The $l_x$ function is given by the period force of mortality at age $x$ ($\mu_x$)

$$l_x = exp(-\int_0^x \mu_a da). \quad (2)$$

In the case of healthy life expectancy (HLE), the survivorship function does not reflect overall survival, but solely survival in good health:

$$HLE_x = \frac{1}{l_x}\int_x^{\infty} l_a^{healthy} da, \quad (3)$$

where $l_x^{healthy}$ defines the probability of surviving in good health from birth to age $x$. This function can be derived from the transition rates between the states "healthy", "unhealthy", and "dead" in a multistate framework. Alternatively, the Sullivan method (1971) offers a shortcut to derive HLE without explicitly deriving $l_x^{healthy}$:

$$HLE_x = \frac{1}{l_x}\int_x^{\infty} \pi_a \cdot l_a \; da, \quad (4)$$

where $\pi_a$ denotes the age-specific proportion of being healthy. The multistate approach is more consistent and produces HLE estimates in a true synthetic cohort fashion, while the Sullivan method combines a synthetic cohort quantity (the mortality trajectory from the period life table population) with prevalence data obtained from the real population. The popularity of the Sullivan method is, therefore, more attributed to lower data requirements (prevalence data vs. transition rates) and less to methodological soundness (see e.g., Laditka and Hayward 2003 for more details). Using the Sullivan method for obtaining the number of healthy life years has important implications for the decomposition of total HLY into group-specific HLYs. Usually, these decomposition methods rely on the fact that mortality rates (or transition rates) which produce the survivorship function (equation 2) are the weighted sum of group-specific rates. This relationship has been shown for example by Shkolnikov et al. (2001) or Torres, Canudas-Romo, and Oeppen (2019).

$$\mu_x = \sum_{i=1}^{N} \mu_x^i p_x^i, \quad (5)$$

with $p_x^i$ being the proportion of the group $i$ in the total population at age $x$. Torres, Canudas-Romo, and Oeppen (2019) have introduced a decomposition technique based on this relationship in order to decompose changes of $e_x$ into the changes in mortality and changes in the population composition. Their approach cannot be simply applied to HLE based on the Sullivan method because of the shortcut described above. However, Shkolnikov et al. (2001) proposed an alternative decomposition which is useful for the aim of this paper. According to Shkolnikov et al. (2001), the sum of the group-specific person-years must be equal to the number of person-years lived by the whole life table population. This holds also for person-years spent in good health produced by the Sullivan method:

$$healthy \; T_x = \sum_{i=1}^{N} healthy \; T_x^i, \quad (6)$$

with healthy $T_x$ being the the number of healthy person years lived from age $x$ to the oldest age. The aim of the decomposition method is finding the life table population weights $\theta_x^i$ that apportion the group-specific person-years "correctly", i.e., their sum should be equal to the total number of person-years. In the simplest case of only two groups, the problem has only one solution. If there are, however, more than two groups the equation allows multiple solutions. For this reason, Shkolnikov et al. (2001) suggest formulating additional constraints, i.e., choosing the weights $\theta_x^i$ that are characterized by a minimum distance from average proportions of groups $i$ in the total population at age $x$ and older ages ($P_{x+}^i/P_{x+}$). This leads

to a problem of minimization with constraints. Mathematically speaking, the problem can be expressed as a system of linear equations. In the case of three groups (i.e., the high-, medium-, and low-educated subpopulations), the expression matrix $A$ and the vector $b$ are:

$$A = \begin{bmatrix} 2 & 0 & 0 & 1 & HLE_x^1 \\ 0 & 2 & 0 & 1 & HLE_x^2 \\ 0 & 0 & 2 & 1 & HLE_x^3 \\ 1 & 1 & 1 & 0 & 0 \\ HLE_x^1 & HLE_x^2 & HLE_x^3 & 0 & 0 \end{bmatrix}, b = \begin{bmatrix} 2(P_{x+}^1/P_{x+}) \\ 2(P_{x+}^2/P_{x+}) \\ 2(P_{x+}^3/P_{x+}) \\ 1 \\ HLE_x \end{bmatrix}, \tag{7}$$

The unknown life table population weights ($\theta_x^i$) can then be obtain from:

$$\boldsymbol{z} = \boldsymbol{A}^{-1} \cdot \boldsymbol{b}. \tag{8}$$

The following example shows how this method can be applied in R.

```
### taking the example of men in Portugal
HLE.low.edu <- 30.55
HLE.medium.edu <- 35.14
HLE.high.edu <- 41.60
HLE.total.edu <- 32.28
### the proportions from the SILC survey (table A1 in the paper)
P.low.edu <- 0.6428
P.medium.edu <- 0.2127
P.high.edu <- 0.1444
### the matrix A
A.m <- matrix(NA, 5, 5)
A.m[1,] <- c(2,0,0,1, HLE.high.edu)
A.m[2,] <- c(0,2,0,1, HLE.medium.edu)
A.m[3,] <- c(0,0,2,1, HLE.low.edu)
A.m[4,] <- c(1,1,1,0,0)
A.m[5,] <- c(HLE.high.edu, HLE.medium.edu, HLE.low.edu, 0, 0)
A.m
```

```
##      [,1]  [,2]  [,3] [,4]  [,5]
## [1,]  2.0  0.00  0.00    1 41.60
## [2,]  0.0  2.00  0.00    1 35.14
## [3,]  0.0  0.00  2.00    1 30.55
## [4,]  1.0  1.00  1.00    0  0.00
## [5,] 41.6 35.14 30.55    0  0.00
```

```
###the b matrix
b.m <- rbind(2*P.high.edu,
             2*P.medium.edu,
             2*P.low.edu,
             1,
             HLE.total.edu
             )
b.m
```

```
##                  [,1]
##                0.2888
##                0.4254
##                1.2856
##                1.0000
## HLE.total.edu 32.2800
```

```r
### getting z
z.m <- solve(A.m) %*% b.m
### the estimated life table population
### weights are
theta.i <- round(z.m[c(1:3),1],2)
cbind(theta.i, c("high", "medium", "low"))
```

```
##      theta.i
## [1,] "0.06"  "high"
## [2,] "0.22"  "medium"
## [3,] "0.71"  "low"
```

In this way, total healthy life expectancy can be expressed as a weighted average of group-specific healthy life expectancies. A more sophisticated approach taking into account the group-specific proportions at each age $x$ has been proposed by Shkolnikov et al. (2001) as well. However, they show that estimates of $\theta_x^i$ do not seriously differ from the simpler procedure. We assume that this holds also for our analysis and, therefore, rely on the more simple version.

## References


- Laditka, S. and Hayward, M. (2003). The Evolution of Demographic Methods to Calculate Health Expectancies. In: Robine, J.M., Jagger, C., Mathers, C.D., Crimmins, E.M., Suzman, R.M. (eds.). Determining Health Expectancies, pp. 221-235.
- Torres, C., Canudas-Romo, V., Oeppen, J. (2019). The contribution of urbanization to changes in life expectancy in Scotland, 1861-1910. Population Studies 73(3), pp. 387-404.
- Shkolnikov, V.M., Valkonen, T., Begun, A., Andreev, E.M. (2001). Measuring inter-group inequalities in length of life. Genus 57, No 3/4, pp. 33-62.
- Sullivan, D.F. (1971). A single index of mortality and morbidity. HSMHA-Health Reports, 86(4), pp. 347-354.

## Calculating $e_x$ and $HLE_x$ with different last open-age intervals

The following R code uses mortality and population data from the Human Mortality Database (HMD). The data can be downloaded at www.mortality.org. We select women in Japan for the sensitivity analysis because they show particularly low mortality levels ($e_0 = 87.49$ in 2019). This low level might be comparable with mortality levels for highly-educated women in European countries. The prevalence data is simulated based on the assumption that the prevalence of being unhealthy increases exponentially with age.

```
setwd("d:/Rcode/Data/")
library(dplyr)

Deaths <- read.table("Japan_Deaths_1x1.txt", header=TRUE, skip=2)
Deaths$Age <- as.numeric(as.character(Deaths$Age))
Deaths$Age[is.na(Deaths$Age)] <- 110

Exposures <- read.table("Japan_Exposures_1x1.txt", header=TRUE, skip=2)
Exposures$Age <- as.numeric(as.character(Exposures$Age))
Exposures$Age[is.na(Exposures$Age)] <- 110

Population <- read.table("Japan_Population.txt", header=TRUE, skip=2)
Population$Age <- as.numeric(as.character(Population$Age))
Population$Age[is.na(Population$Age)] <- 110

mx.function <- function(Deaths, Exposure, the.year, openage) {

    Deaths.year <- filter(Deaths, Year==the.year)
    Expo.year <- filter(Exposures, Year==the.year)
    last.age.i <- openage+1

    Deaths.year.sum.f <- c(Deaths.year$Female[1:openage],
                           sum(Deaths.year$Female[c(last.age.i:111)])
                           )

    Expo.year.sum.f <- c(Expo.year$Female[1:openage],
                           sum(Expo.year$Female[c(last.age.i:111)])
                           )

    Deaths.year.sum.m <- c(Deaths.year$Male[1:openage],
                           sum(Deaths.year$Male[c(last.age.i:111)])
                           )

    Expo.year.sum.m <- c(Expo.year$Male[1:openage],
                           sum(Expo.year$Male[c(last.age.i:111)])
                           )

    mx.f <- Deaths.year.sum.f/Expo.year.sum.f
    mx.m <- Deaths.year.sum.m/Expo.year.sum.m
    out <- data.frame(Age=0:openage, Female=mx.f, Male=mx.m)

    return(out)
}

life.table <- function(mx){
    ax <- c(0.14, rep(0.5, length(mx)-1))
    qx <- mx/(1+(1-ax)*mx)
```

```
    qx[length(qx)] <- 1
    qx[qx > 1] <- 1
    px <- 1-qx
    lx <- c(100000, (cumprod(px)*100000)[1:(length(px)-1)])
    dx <- c(-diff(lx), lx[length(lx)])
    Lx1 <- lx[-1]+ax[-length(ax)]*dx[-length(dx)]
    open.Lx <-  ifelse( mx[length(mx)] == 0, 0, dx[length(dx)]/mx[length(mx)])
    Lx <- c(Lx1, open.Lx)
    Tx <- rev(cumsum(rev(Lx)))
    ex <- Tx/lx

    return(data.frame(qx=qx, px = px, ax = ax,
                      lx = lx , dx = dx, Lx= Lx,
                      Tx = Tx, ex = ex))
}

Prev.function <- function(Population, the.year, openage) {

    Pop.year <- filter(Population, Year==the.year)
    proportions <- exp(seq(log(0.05), log(0.95), length.out=111))
    Unhealthy.Pop.f <- Pop.year$Female*proportions
    Unhealthy.Pop.m <- Pop.year$Male*proportions

    last.age.i <- openage+1

    Pop.year.sum.f <- c(Pop.year$Female[1:openage],
                           sum(Pop.year$Female[c(last.age.i:111)])
                           )

    Pop.year.sum.m <- c(Pop.year$Male[1:openage],
                           sum(Pop.year$Male[c(last.age.i:111)])
                           )

    Unhealthy.Pop.sum.f <- c(Unhealthy.Pop.f[1:openage],
                           sum(Unhealthy.Pop.f[c(last.age.i:111)])
                           )

    Unhealthy.Pop.sum.m <- c(Unhealthy.Pop.m[1:openage],
                           sum(Unhealthy.Pop.m[c(last.age.i:111)])
                           )

    prev.f <- Unhealthy.Pop.sum.f/Pop.year.sum.f
    prev.m <- Unhealthy.Pop.sum.m/Pop.year.sum.m

    out <- data.frame(Age=0:openage, Female=prev.f, Male=prev.m)

    return(out)
    }


final.function <- function(LT.out, Prev.out) {

    Sulli <- LT.out
```

```r
    Sulli$Prev <- Prev.out
    Sulli$Lx.h <- Sulli$Lx*(1-Sulli$Prev)
    Sulli$Tx.h <- rev(cumsum(rev(Sulli$Lx.h)))
    Sulli$HLY  <- Sulli$Tx.h/Sulli$lx

    return(Sulli)
}

###Analysis for Japanese women in 2019

###with age 80+
mx.80 <- mx.function(Deaths, Exposures, 2019, 80)
prev.80 <- Prev.function(Population, 2019, 80)
LT.out <- life.table(mx.80$Female)
Sulli <- final.function(LT.out, prev.80$Female)

ex.and.HLY.80 <- data.frame(ex=Sulli$ex, HLY=Sulli$HLY)

###with age 85+
mx.85 <- mx.function(Deaths, Exposures, 2019, 85)
prev.85 <- Prev.function(Population, 2019, 85)
LT.out <- life.table(mx.85$Female)
Sulli <- final.function(LT.out, prev.85$Female)

ex.and.HLY.85 <- data.frame(ex=Sulli$ex, HLY=Sulli$HLY)

###with age 95+
mx.95 <- mx.function(Deaths, Exposures, 2019, 95)
prev.95 <- Prev.function(Population, 2019, 95)
LT.out <- life.table(mx.95$Female)
Sulli <- final.function(LT.out, prev.95$Female)

ex.and.HLY.95 <- data.frame(ex=Sulli$ex, HLY=Sulli$HLY)

###with age 110+
mx.110 <- mx.function(Deaths, Exposures, 2019, 110)
prev.110 <- Prev.function(Population, 2019, 110)
LT.out <- life.table(mx.110$Female)
Sulli <- final.function(LT.out, prev.110$Female)

ex.and.HLY.110 <- data.frame(ex=Sulli$ex, HLY=Sulli$HLY)

###plot the prevalence data
par(mfrow=c(2,2))
plot(0:80, 1-prev.80$Female, type="l", main="Last open-age at 80 years",
     xlab="Age", ylab="Prevalence")
plot(0:85, 1-prev.85$Female, type="l", main="Last open-age at 85 years",
     xlab="Age", ylab="Prevalence")
plot(0:95, 1-prev.95$Female, type="l", main="Last open-age at 95 years",
     xlab="Age", ylab="Prevalence")
plot(0:110, 1-prev.110$Female, type="l", main="Last open-age at 110 years",
     xlab="Age", ylab="Prevalence")
```

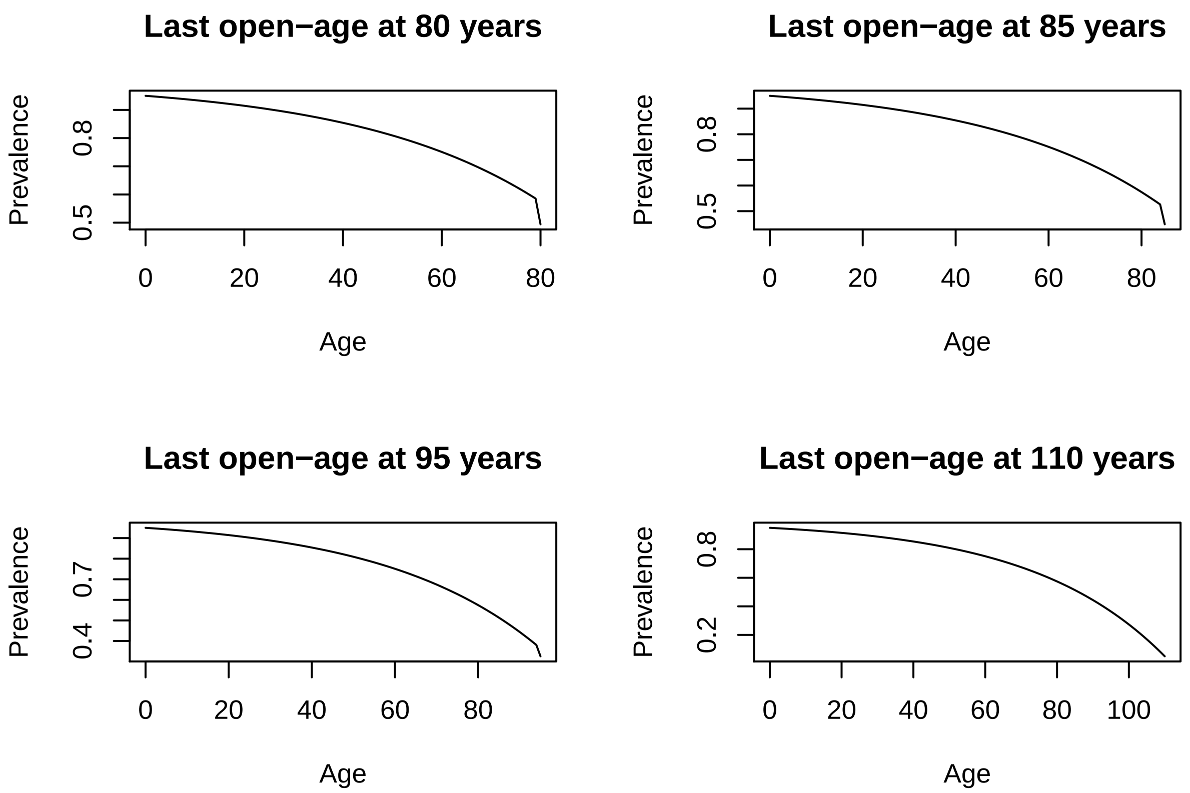


```
###comparing e0 and HLY0

rbind(ex.and.HLY.80[1,],
      ex.and.HLY.85[1,],
      ex.and.HLY.95[1,],
      ex.and.HLY.110[1,])
```

```
##         ex      HLY
## 1 89.13272 70.18604
## 2 88.29782 69.68190
## 3 87.55151 69.27327
## 4 87.49727 69.24647
```

```
###comparing e30 and HLY30

rbind(ex.and.HLY.80[31,],
      ex.and.HLY.85[31,],
      ex.and.HLY.95[31,],
      ex.and.HLY.110[31,])
```

```
##           ex      HLY
## 31  59.60388 42.81681
## 311 58.76383 42.30957
## 312 58.01292 41.89841
## 313 57.95834 41.87144
```

```
###comparing e50 and HLY50
```

```r
rbind(ex.and.HLY.80[51,],
      ex.and.HLY.85[51,],
      ex.and.HLY.95[51,],
      ex.and.HLY.110[51,])
```

```
##            ex      HLY
## 51  40.20818 26.14013
## 511 39.35731 25.62635
## 512 38.59672 25.20990
## 513 38.54144 25.18258
```

The results indicate that the choice of the last open-age interval has a smaller impact on HLY compared to $e_x$. In general, the impact on HLY is relatively small and will most likely not change the country ranking of (education-adjusted) HLY across Europe substantially.

## Investigating and evaluating the education-specific mortality data from Eurostat

The following R code provides education-specific $e_30$ estimates over time. This helps understanding the reliability and robustness of these estimates. The corresponding plots are shown at the end of the document.

```
library(eurostat)
library(dplyr)

data <- get_eurostat("demo_mlexpecedu", time_format = "num")

#rename and redefine the file
data$isced11 <- as.character(data$isced11)
data$isced11 <- ifelse(data$isced11=="ED0-2", "lower", data$isced11)
data$isced11 <- ifelse(data$isced11=="ED3_4", "middle", data$isced11)
data$isced11 <- ifelse(data$isced11=="ED5-8", "higher", data$isced11)
data$isced11 <- ifelse(data$isced11=="TOTAL", "total", data$isced11)

data$age <- as.character(data$age)
data$age <- ifelse(data$age=="Y_LT1", "Y0", data$age)
data$age <- ifelse(data$age=="Y_GE85", "Y85", data$age)
data$age <- substring(data$age, 2)

data <- data[,-1]
colnames(data) <- c("sex","age","edu","country","year","ex")
data$age <- as.numeric(data$age)

edu.countries <- c("BG","DK","EE","EL","HR","IT","HU", "CZ",
                   "PL","PT","RO","SI","SK","FI","SE","NO")

data <- filter(data, country %in% edu.countries)
data.age <- filter(data, age==30, sex=="T")
data.age <- arrange(data.age, year)
```

In general, the Nordic countries (Denmark, Norway, Finland, and Sweden) appear as being most reliable. While the remaining countries show substantial fluctuations over time, the $e_x$ values for the Nordic countries are more robust. Looking at the age-standardized mortality rates (ASMR) presented by Mackenback et al. (2018) might provide further evidence for their reliability. Even though $e_x$ cannot uncritically compared with ASMR, both mortality measures show similar mortality levels, i.e., Sweden shows lowest mortality, Denmark's level is slightly higher, and Finland and Norway are falling somewhere in between. The exact reasons for the high fluctuations in some countries are not clear. According to Eurostat (2015), the highest level of educational attainment is registered on the death declaration in Bulgaria, Czech Rep. (on valontary basis), Estonia, Greece, Croatia, Italy, Hungary, Poland, Portugal, Romania, and Slovakia. Moreover, Corsini (2010) notes that the information on educational attainment for Bulgaria, Czech Rep., Estonia, Italy, Hungary, Poland, Romania, and Slovenia has been derived from the European Labour Force Survey. She further states, that these are experimental statistics which will be developed further in the future and any conclusions should be drawn with caution. As this article was published already in 2010, some of the fluctuations might be explained by changes in the estimation method used by Eurostat. In addition, changes to educational attainment classification might have affected the education-specific mortality over time. Eurostat used ISCED 1997 before 2014, which it replaced with ISCED 2011 afterwards. Looking at the $e_30$ time trend reveals that this classification change is associated with a drop in $e_30$ for the highly educated subpopulation, while $e_30$ increased for some analyzed countries in the low-educated group. This might explain why we observe higher $e_30$ levels for low- or medium-educated individuals compared to highly educated persons in some countries. Mackenbach et al. (2018) used harmonized data and find that the mortality levels for the high-educated subpopulation is consistently lower compared to the low-educated subpopulation in all analyzed countries. To sum up the evaluation of education-specific mortality data by Eurostat, the presented are likely to be

affected by (1) differences in the data collection, (2) changes in the method for deriving education-specific estimates, (3) changes in the classification of educational attainment. Data for Nordic countries seem more reliable compared to the remaining countries. Readers should be aware of these data limitations and interpret the presented results with caution.

## References


- Corsini, V. (2010). Highly educated men and women likely to live longer: Life expectancy by educational attainment. Eurostat - Statistics in focus 24/2010, Population and social conditions.
- Eurostat (2015). Demographic statistics: A review of definitions and methods of collection in 44 European countries.
- Mackenbach, J. P., Valverde, J. R., Artnik, B., Bopp, M., Brønnum-Hansen, H., Deboosere, P., Kalediene, R., Kovács, K., Leinsalu, M., Martikainen, P., Menvielle, G., Regidor, E., Rychtaříková, J., Rodriguez-Sanz, M., Vineis, P., White, C., Wojtyniak, B., Hu, Y., and Nusselder, W. J. (2018). Trends in health inequalities in 27 European countries. Proceedings of the National Academy of Sciences of the United States of America, 115(25), pp. 6440–6445.


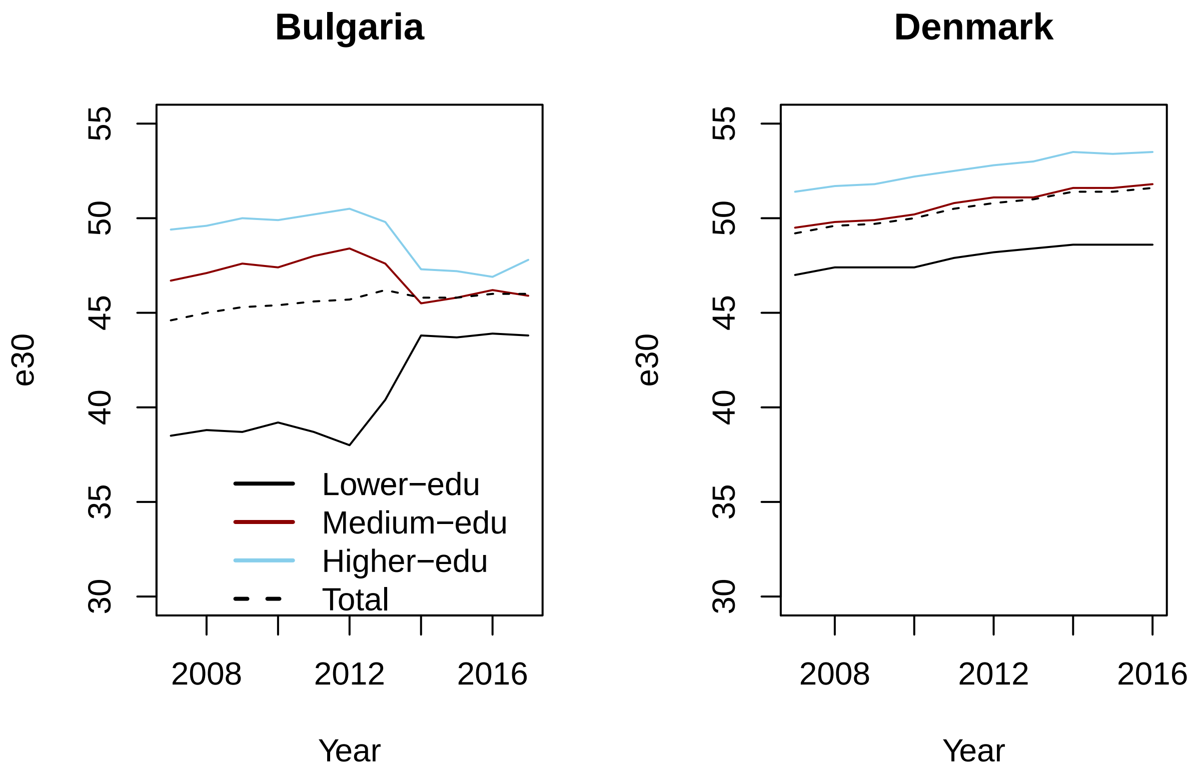

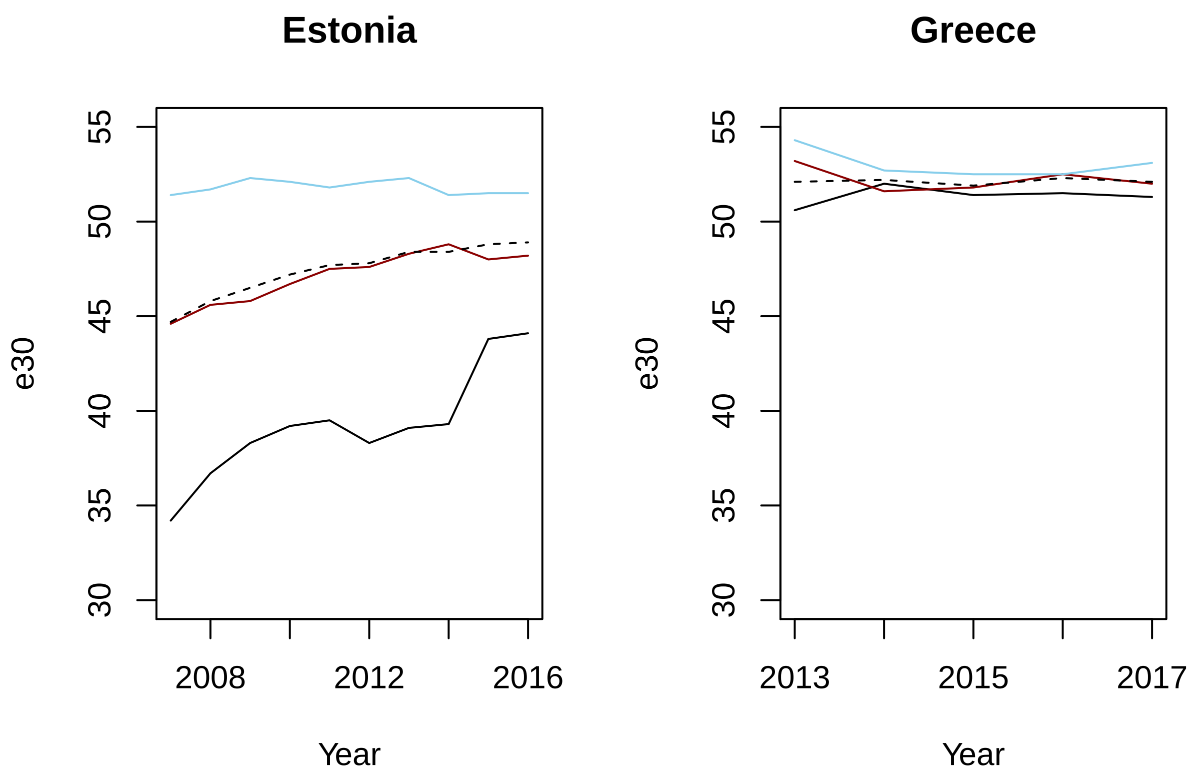
Estonia
Greece
e30
e30
55
50
45
40
35
30
2008
2012
2016
2013
2015
2017
Year
Year

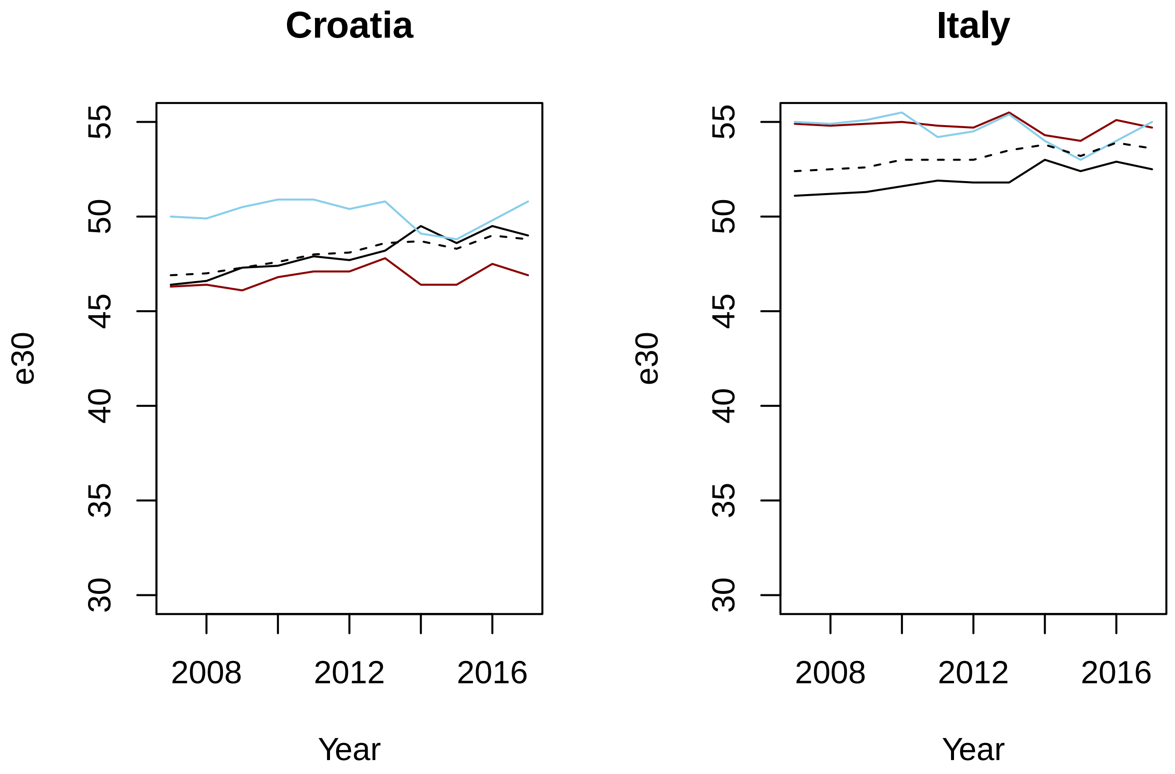

Croatia
e30
55
50
45
40
35
30
2008
2012
2016
Year
Italy
e30
55
50
45
40
35
30
2008
2012
2016
Year

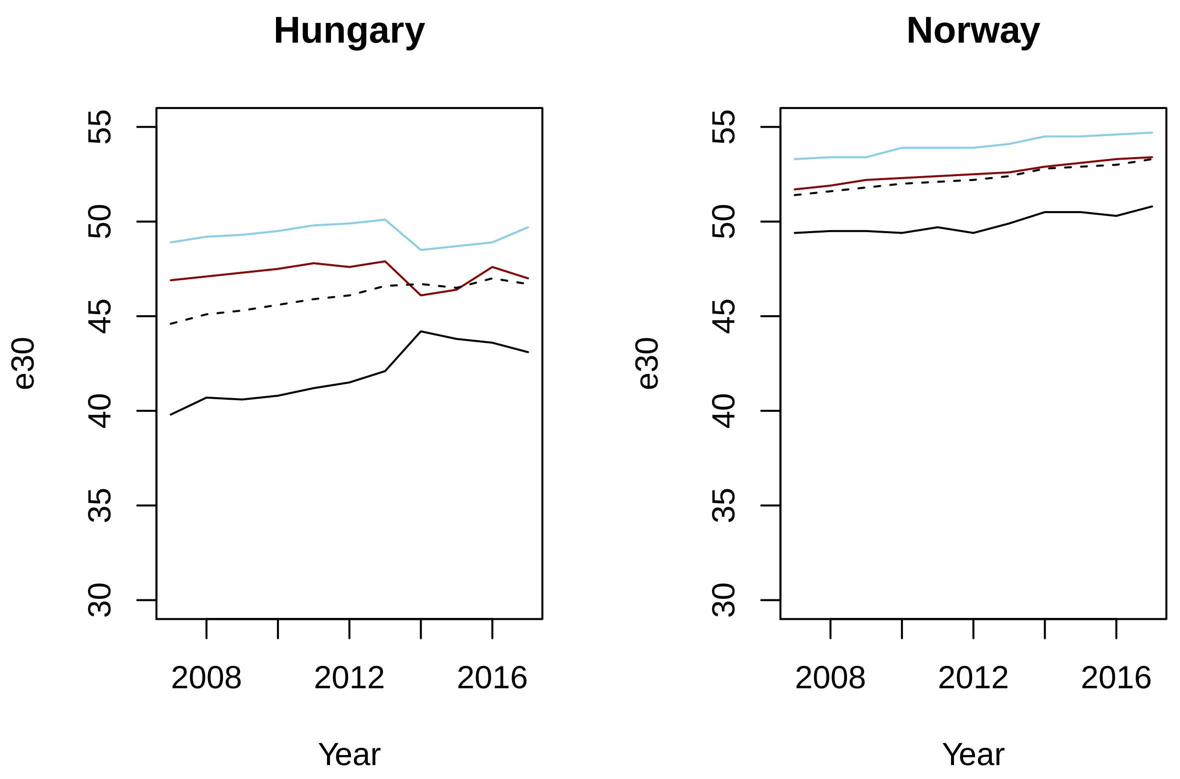
Hungary
Norway
e30
55
50
45
40
35
30
2008
2012
2016
Year

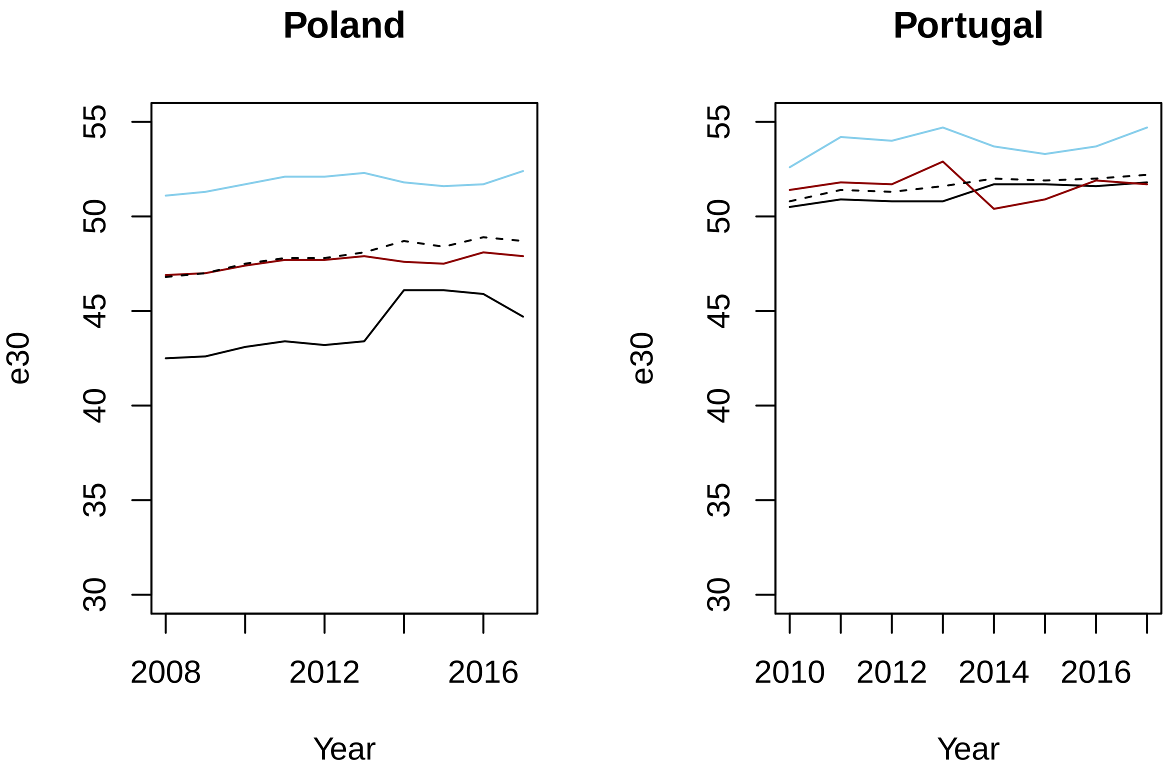

Poland
Portugal
e30
e30
55
50
45
40
35
30
2008
2012
2016
2010
2012
2014
2016
Year
Year

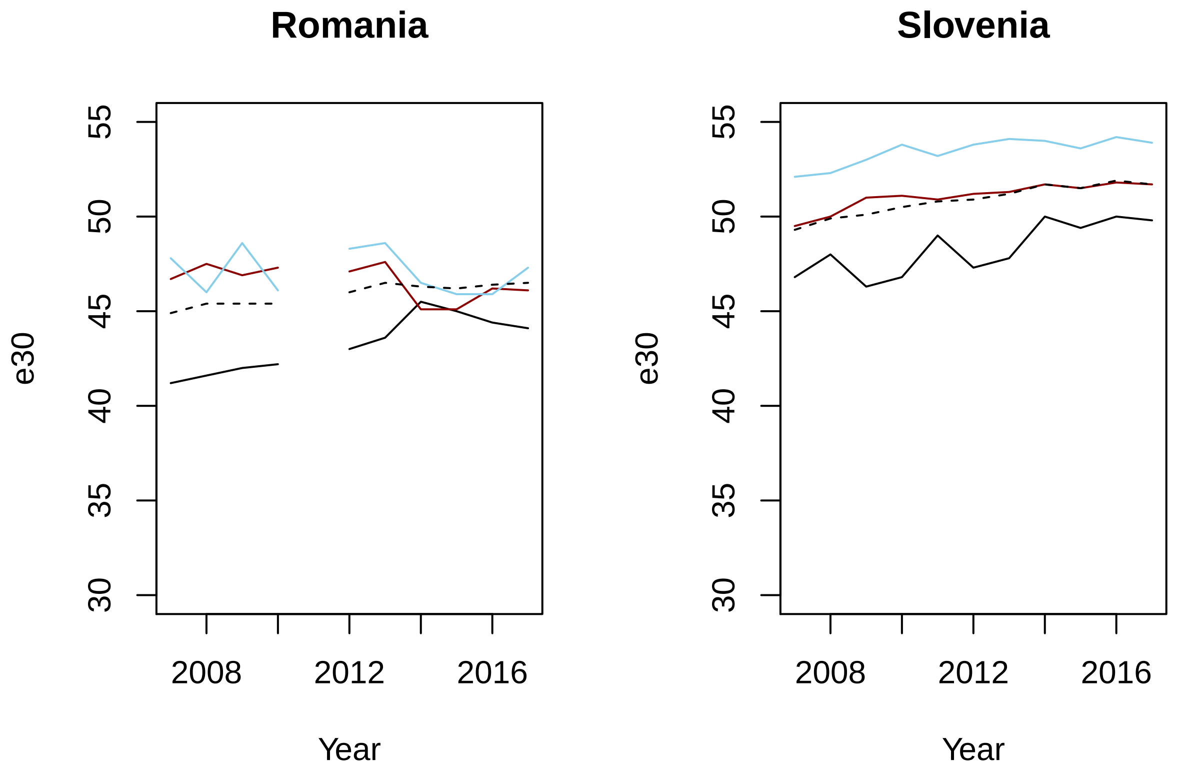
Romania
Slovenia
e30
55
50
45
40
35
30
2008
2012
2016
Year

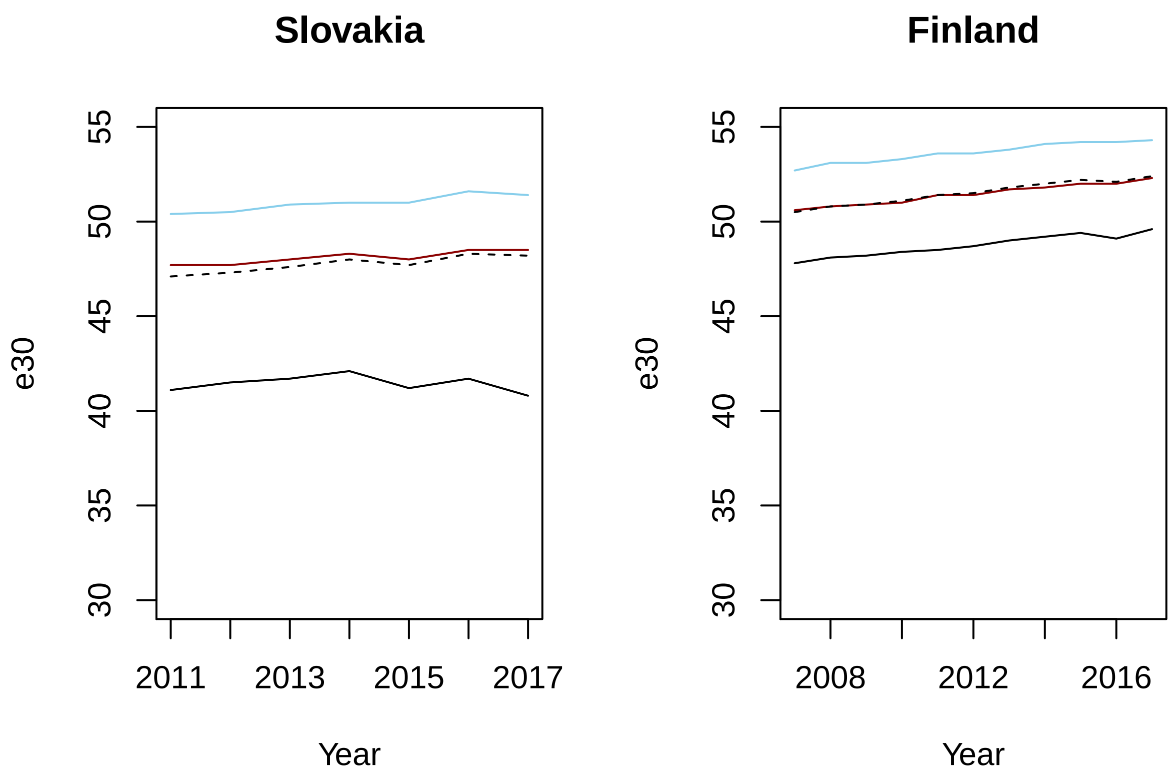

Slovakia
Finland
e30
55
50
45
40
35
30
2011
2013
2015
2017
2008
2012
2016
Year

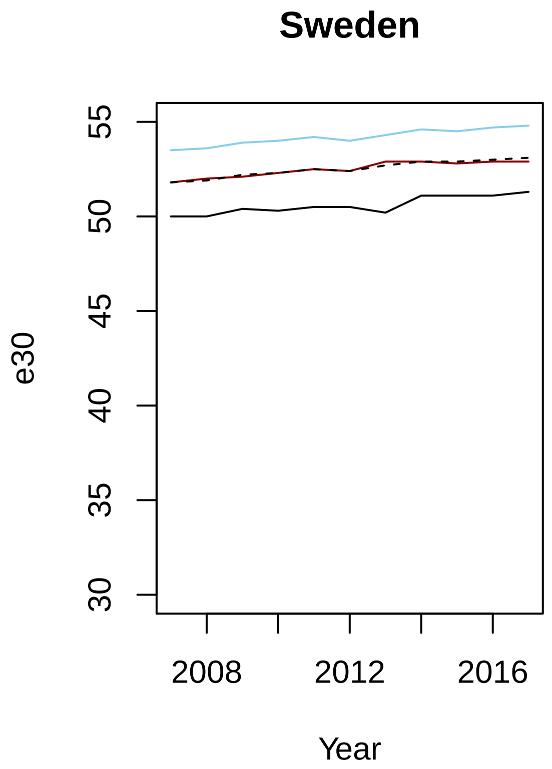

Sweden
e30
55
50
45
40
35
30
2008
2012
2016
Year
